# Participatory Action Design and Engineering of Powered Personal Transfer System for Wheelchair Users: Initial Design and Assessment

**DOI:** 10.3390/s23125540

**Published:** 2023-06-13

**Authors:** Shantanu A. Satpute, Jorge Luis Candiotti, Jonathan A. Duvall, Hailee Kulich, Rosemarie Cooper, Garrett G. Grindle, Benjamin Gebrosky, Josh Brown, Ian Eckstein, Sivashankar Sivakanthan, Nikitha Deepak, Joshua Kanode, Rory A. Cooper

**Affiliations:** 1 Human Engineering Research Laboratories, School of Health and Rehabilitation Sciences, Pittsburgh, PA 15206, USA; shs220@pitt.edu (S.A.S.); jlc118@pitt.edu (J.L.C.); jad75@pitt.edu (J.A.D.); hrk6@pitt.edu (H.K.); cooperrm@pitt.edu (R.C.); ggg3@pitt.edu (G.G.G.); gebrosky@pitt.edu (B.G.); jdb83@pitt.edu (J.B.); ije7@pitt.edu (I.E.); sis65@pitt.edu (S.S.); nid51@pitt.edu (N.D.); jok123@pitt.edu (J.K.); 2 Human Engineering Research Laboratories, VA Pittsburgh Healthcare System, Pittsburgh, PA 15206, USA; 3 Department of Bioengineering, Swanson School of Engineering, University of Pittsburgh, Pittsburgh, PA 15261, USA

**Keywords:** caregivers, injury prevention, assistive technology, patient transfer device, rehabilitation robotics, wheeled mobility, disability

## Abstract

Caregivers that assist with wheelchair transfers are susceptible to back pain and occupational injuries. The study describes a prototype of the powered personal transfer system (PPTS) consisting of a novel powered hospital bed and a customized Medicare Group 2 electric powered wheelchair (EPW) working together to provide a no-lift solution for transfers. The study follows a participatory action design and engineering (PADE) process and describes the design, kinematics, and control system of the PPTS and end-users’ perception to provide qualitative guidance and feedback about the PPTS. Thirty-six participants (wheelchair users (n = 18) and caregivers (n = 18)) included in the focus groups reported an overall positive impression of the system. Caregivers reported that the PPTS would reduce the risk of injuries and make transfers easier. Feedback revealed limitations and unmet needs of mobility device users, including a lack of power seat functions in the Group-2 wheelchair, a need for no-caregiver assistance/capability for independent transfers, and a need for a more ergonomic touchscreen. These limitations may be mitigated with design modifications in future prototypes. The PPTS is a promising robotic transfer system that may aid in the higher independence of powered wheelchair users and provide a safer solution for transfers.

## 1. Introduction

An estimated 5.5 million Americans, including 250,000 veterans rely on wheelchairs for mobility [1,2]. Many of them need assistance from caregivers for activities of daily living, including transferring from a wheelchair to a bed and vice-versa. However, personal transfers can be physically demanding and involve a moderate risk of injury for wheelchair users as well as caregivers.

According to the Bureau of Labor Statistics, caregivers have the highest incidence of nonfatal occupational injury [3]. About 74–90% of professional caregivers and informal caregivers (family and friends) report musculoskeletal injuries and pain [4,5,6,7]. Assisting with transfers, even when using mechanical assist devices, involves frequent lifting and repositioning, which has been shown to exceed the safe limits for forces on the back [8,9,10]. Previous research has reported that 43% of musculoskeletal injuries in caregivers are a result of assistance with transfer-related activities [11]. Besides adverse physical effects, about 45% of caregivers attending to people with long-term disability experience emotional strain, which may hamper their ability to perform transfer tasks safely [12,13].

Several transfer systems have been researched and developed to mitigate the risk of injury to caregivers and their care recipients, as highlighted in a recent literature review [14]. While slide boards are commonly used to reduce arm forces, they are still physically demanding and may not significantly reduce the risk of injury [15]. Clinical settings may be equipped with lift devices but are often inconvenient or time-inefficient [16]. Mechanical lifts such as portable gantry lifts and ceiling-mounted lifts require lower arm forces and reduce stress at the lumbosacral joint (L5/S1) [17]. However, they require the caregiver to position a sling under the care recipient, which may include lifting, repositioning, and sliding the care recipient, still making it physically demanding [18]. Whilst using mechanical lifts, the care recipient is suspended in the air, which exposes them to anxiety and the risk of falls, potentially leading to injuries and trauma [19]. Additionally, ceiling-mounted lifts are not always practical due to the requirement of complex installation and architectural design or modifications [16]. There is a paucity of practical and reliable person transfer systems and a need for user-centered design and development of superior transfer technologies [20]. A recent survey of over 1000 mobility device users highlighted the need for better transfer devices as an area of critical importance to care recipients [2].

Robotic transfer systems are emerging to mitigate the limitations and risks associated with mechanical lifts [21,22]. However, robotic transfer systems have not been developed or researched extensively [14]. The AgileLife patient transfer system (PTS), developed by NextHealth LLC [23], is a commercial robotic transfer that consists of a customized bed and a customized manual wheelchair to provide a no-lift solution for transfers between these devices. Previous research consisting of a preliminary evaluation of the PTS reported a positive impact on caregivers and care recipients [24]. Both wheelchair users and their caregivers reported a significant reduction in physical demand and work after six weeks of use. The PTS was rated more favorably concerning safety, functionality, and ease of use compared to other transfer systems by rehabilitation professionals. However, the commercially available PTS is limited to those who can use a manual wheelchair for mobility.

To expand the target population, our study built upon the PTS bed to interface with an electric power wheelchair (EPW). The proposed powered personal transfer system (PPTS) is intended for Medicare Group 2 EPW (powered wheelchair with programmable controller and a captain seat) users. The system utilizes sensors and a control system for the bed and EPW, along with a custom-designed seat and backrest for the EPW, which provides a no-lift solution.

The objectives of this study were (1) to describe the hardware, kinematics of motion, and control system framework of the PPTS and (2) to gather qualitative feedback about the PPTS prototype from EPW users and caregivers via focus groups. The development and evaluation of the PPTS followed a participatory action design and engineering (PADE) process, where end-users (key stakeholders) are involved in every aspect of research and development for the eventual successful implementation and adoption of assistive technologies [25,26]. The engagement of end-users was crucial to understanding the needs for the system, perception of the prototype, and identifying future areas to address, which serve as a pathway for further development and modifications in the iterative design process.

## 2. Materials and Methods

### 2.1. The Description of the PPTS

#### 2.1.1. Overview

The prototype of PPTS was developed using a modified version of the Agilelife patient transfer system (PTS) bed and a modified Medicare Group 2 powered wheelchair (Jazzy Elite ES-1; distributed by Pride Mobility, Duryea, PA, USA) [27]. The modifications for the Medicare Group 2 powered wheelchair required that the chair and bed both follow a similar set of motions to the original Agilelife PTS. To facilitate the transfer, the following engineering criteria were identified:

- Inhibit EPW driving mode during transfer;
- The EPW backrest must be displaced to clear the path between EPW and the bed;
- The EPW seat pan must move along with the bed’s leg segment to safely transfer the person (i.e., maintain the user’s posture/comfort);
- The PPTS will follow a finite-state machine model with checkpoints to ensure a safe transfer process (sensor feedback).

#### 2.1.2. The Robotic Bed

**Figure 1:**
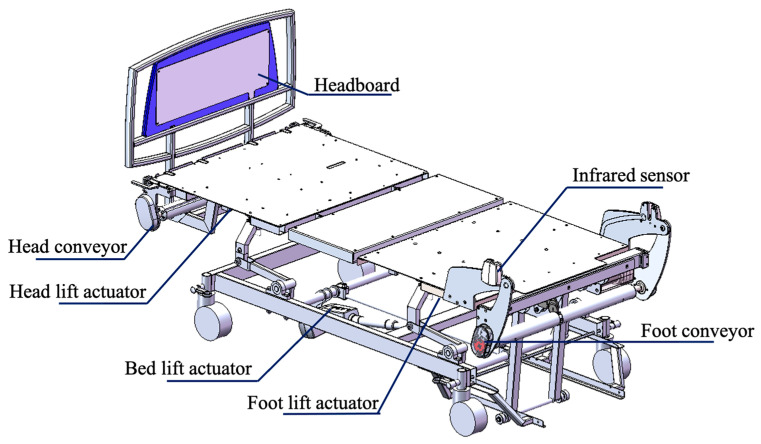
CAD model of the PPTS Bed.

The PPTS bed is similar to a hospital bed (Figure 1), with additional functionalities to facilitate a person’s transfer. It consists of three DC linear actuators that individually raise/lower the head portion of the bed (referred to as headrest), the leg portion of the bed (leg rest) and the bed height. Additionally, the bed includes two rotary actuators, each positioned at the head and foot of the bed, that work in sync to drive a conveyor sheet placed over the mattress. The conveyor sheet moves either towards or away from the headboard to reposition the user in bed.

**Figure 2:**
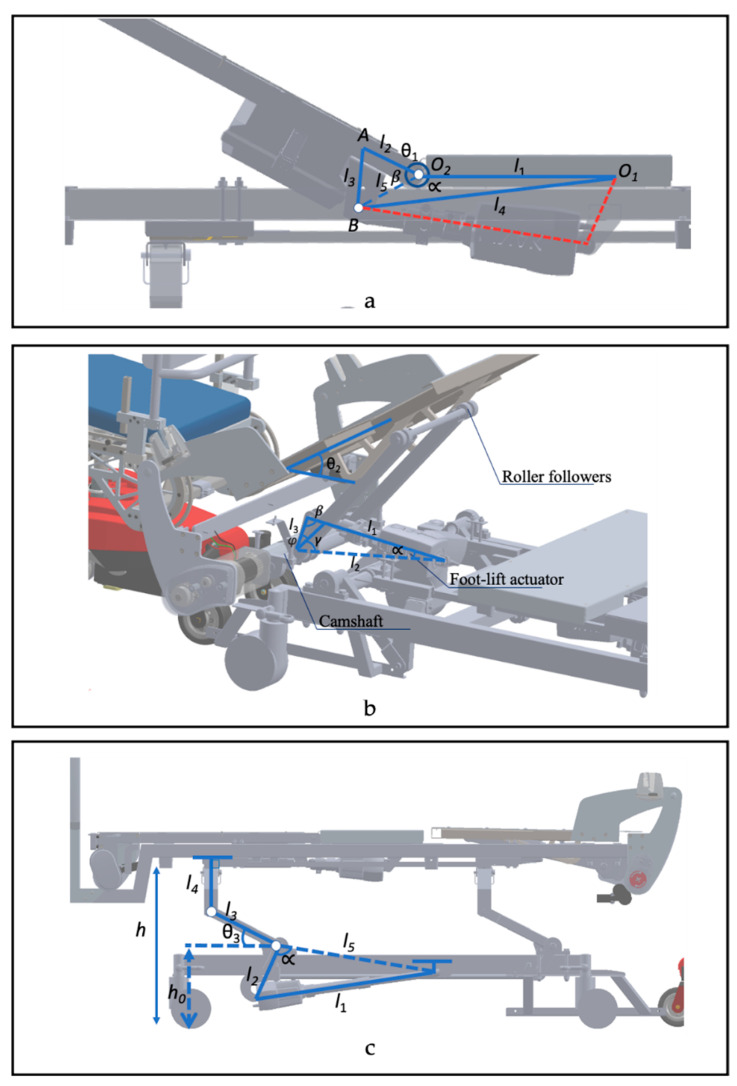
Kinematics of the PPTS Bed. (**a**) Head-lift mechanism. (**b**) Foot-lift mechanism. (**c**) Bed-lift mechanism.

The headrest of the bed mechanism uses a four-bar linkage (O_1_O_2_AB) such that the amount of backrest lift (*θ*_1_) is controlled by the actuator stroke (*l*_4_) (Figure 2a). It is designed to tilt between 0° and 60°, allowing users to go from supine to Fowler’s positions. The relationship between the linear actuator stroke and angle *θ*_1_ (in radians) is described by the following equation using cosine laws and trigonometric identities.

$$\theta_{1}=2\pi -\cos^{-1}\left(\frac{l_{5}^{2}+l_{1}^{2}-l_{4}^{2}}{2l_{1}l_{5}}\right)-\cos^{-1}\left(\frac{l_{2}^{2}+l_{5}^{2}-l_{3}^{2}}{2l_{2}l_{5}}\right)$$

The bed leg rest is designed to tilt using a cam-follower mechanism. It is designed such that the linear actuator rotates the camshaft, as shown in Figure 2b. As the actuator stroke ($l_{1}$) increases, the roller-followers rotate the leg rest plate at a *θ*_2_ angle. The relationship between the actuator stroke and *θ*_2_ is given by the following equations.

$$\theta_{2}=\pi -\phi -\cos^{-1}\left[\frac{l_{3}^{2}+l_{1}^{2}-l_{2}^{2}}{2l_{1}l_{3}}\right]+\frac{13\pi }{180}\;\;\;\;\;\;\;\;[\frac{\pi }{12}<\alpha <\frac{2\pi }{9}]$$

$$\theta_{2}=\phi_{0}=\frac{11\pi }{90}\;\;\;\;\;\;\;\;\;\;[0<\alpha <\frac{\pi }{12}]$$

$$\theta_{2}=0.223l_{1}-8.23\;\;\;\;\;\;\;\;\;[\alpha >\frac{\pi }{12}]\;$$

The bed height is controlled by a linear actuator that is linked to a camshaft, such that as the actuator extends, the camshaft is rotated in a counter-clockwise direction (Figure 2c). This mechanism increases angle *θ*_3_, leading to an increase in the height of the bed (h). The height of the bed is given by the following equations, where $\alpha^{0}$ and $\theta_{3}^{0}$ are the initial constant angles (corresponding to angles α and $\theta_{3},$ respectively) when the height of the bed is in its lowest position (i.e., when $h=h_{0})$.

$$h=h_{0}+l_{3}\sin\theta_{3}+l_{4}$$

$$\theta_{3}=\cos^{-1}\left(\frac{l_{5}^{2}+l_{2}^{2}-l_{1}^{2}}{2l_{5}l_{2}}\right)-\alpha^{0}-\theta_{3}^{0}$$

#### 2.1.3. The Chair

**Figure 3:**
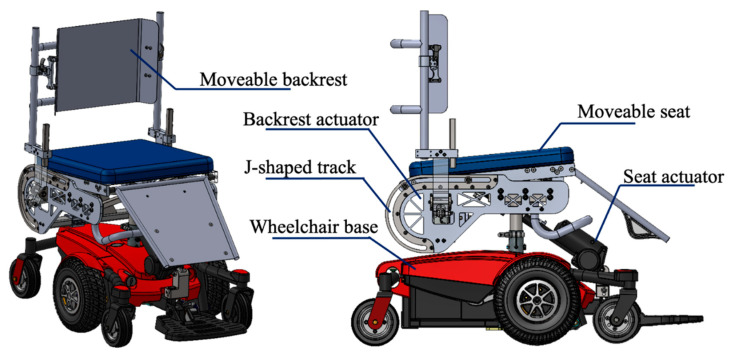
CAD model of the customized EPW.

The original equipment manufacturers’ (OEM) seating system was removed from the wheelchair to incorporate a custom seating system as described below (Figure 3). The custom EPW seating system consists of two linear actuators to independently slide the backrest away from the EPW footprint and tilt the seat pan.

**Figure 4:**
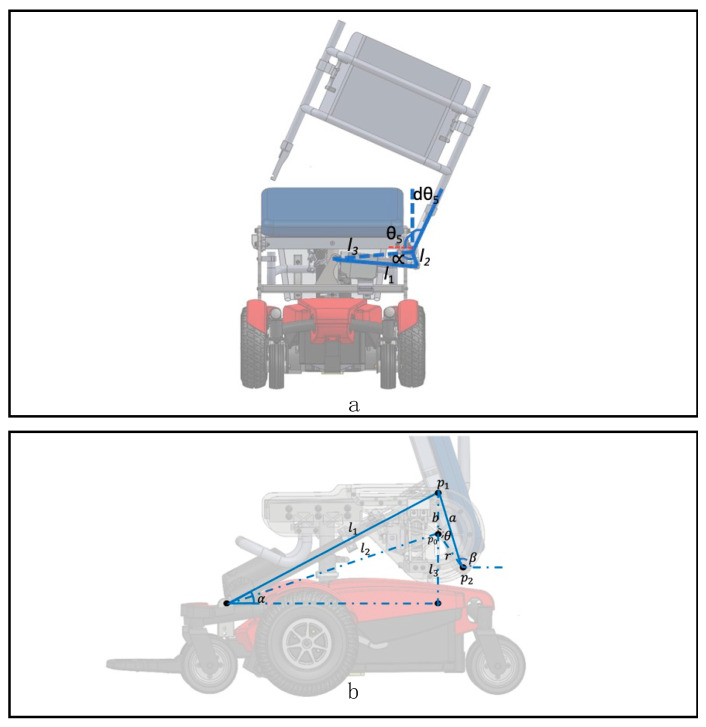
Kinematics of the EPW. (**a**) Backrest slide mechanism. (**b**) Seat rotation mechanism.

The backrest actuator (LA23, Linak, Louisville, KY, USA) was attached to the frame of the seating system and its other end to a cantilever bar on the side of the seat frame to translate its linear stroke ($l_{1}$) to the backrest angle (*dθ*_5_). (Figure 4a). This mechanism allows the backrest to be displaced ($l_{1})$ up to 100° to the side of the EPW. The relationship between the actuator stroke and a backrest angle is described in the following equation derived by cosine laws:

$$d\theta_{5}=\theta_{5}-\cos^{-1}\left(\frac{l_{3}^{2}+l_{2}^{2}-l_{1}^{2}}{2l_{3}l_{2}}\right)$$

A second linear actuator (LA30, Linak, KY) is attached to the EPW base frame, and a bracket on the underside of the seat pan (Figure 4b). As the actuator extends, the seating pan moves along a “J” shaped track (semicircle with radius = *r*) attached to both sides of the EPW. The ”J” shaped track allows the seat pan to tilt back up to 90°. This brings the seat pan closer to the bed, minimizing the gap between the chair and the bed during a transfer, and brings the user’s legs and feet toward the bed with minimal shearing. The seat pan angle (β) is obtained from two cartesian coordinates points of the seat pan ($p_{1}$,$\;p_{2}$). These points move along with the ‘J-track’ mechanism and the actuator stroke (*l*). $p_{1}$ coordinates are obtained by the angle between the actuator and a horizontal plane, while $p_{2}$ coordinates are a function of Δθ (change in θ). θ is the cross angle between vectors $p_{01}$ and $p_{02}$ and obtained using cosine law. The equations for the kinematics are given below.

$$p_{1}=f\left(\alpha \right);\;\alpha =\sin^{-1}\left(\frac{l_{3}+r}{l}\right)$$

$$p_{2}=f\left(\Delta \theta \right);\Delta \theta =\theta -\theta_{0}$$

$$\theta_{0}=\cos^{-1}\left(\frac{b_{0}^{2}+r^{2}-a^{2}}{2*b*r}\right)$$

$$\beta =180-\tan^{-1}\left[\frac{\Delta p_{y}}{\Delta p_{x}}\right]$$

$$p_{1}=f\left(\alpha \right);$$

$$p_{2}=f\left(\Delta \theta \right)$$

$$\Delta \theta =\cos^{-1}\left(\frac{\Delta p_{01}^{2}+p_{02}^{2}-p_{12}^{2}}{2\times \Delta p_{01}\times p_{02}}\right)$$

$$\beta =180-\tan^{-1}\left[\frac{\Delta p_{y}}{\Delta p_{x}}\right]$$

#### 2.1.4. Cyberphysical System and Control

**Figure 5:**
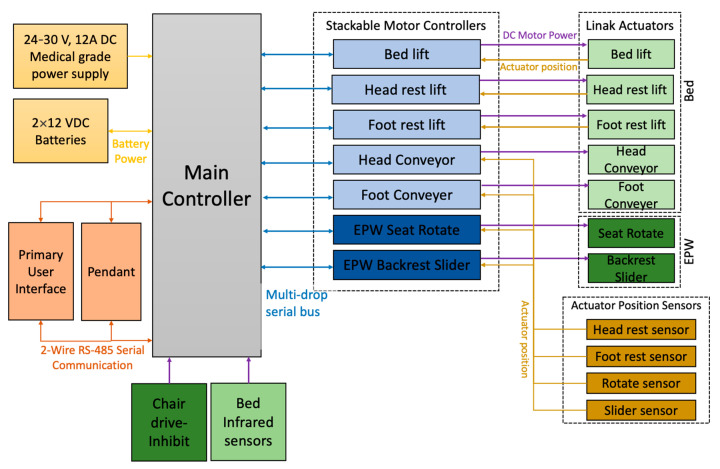
Block diagram for PPTS system.

The PPTS is powered by a 24–30 V, 12A medical-grade power supply. Additionally, two 12 Volt DC batteries connected in series are included to power the system in case of a power outage. The PPTS system is equipped with 7 linear actuators, 5 of which are embedded in the bed (shown in light green in Figure 5) and 2 in the custom EPW seat (shown in dark green in Figure 5). Each actuator includes a position sensor to monitor the motion of the bed and EPW linkages. The EPW seat pan and backrest actuators are powered and controlled from the bed using a wired connection. The main controller communicates with the seven motor controllers via a multidrop serial bus. The main controller checks the activity of each motor controller every 100 ms. Active motor controllers are polled every 100 ms to check for a received response. The motor controller then communicates the status of a given actuator to the main controller. This ensures that the main controller is aware of the status of each actuator at each sampling time. The PPTS bed is equipped with infrared sensors to sense the position of the person.

**Figure 6:**
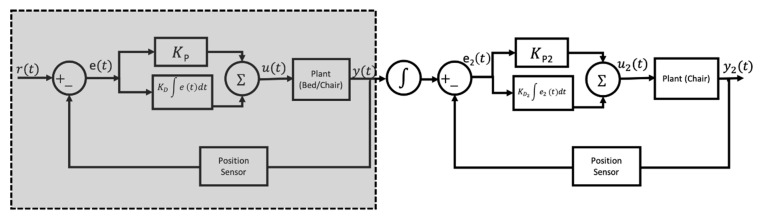
PI controller(s) for PPTS.

Each motor controller moves its corresponding actuator to a desired linear speed *y(t)* by feeding the difference between the desired *r_d(t)_* and actual position/orientation *r_a(t)_* of each bed/EPW linkage to a classic proportional-integrate (PI) controller *u(t)* (Figure 6 (shaded)).

During the transfer process, the bed leg rest rises as the EPW backrest swings away, such that the bed leg rest serves as a reclined backrest for the person being transferred. Next, the bed headrest and EPW seat pan rotate synchronously to bring the user’s legs towards the bed and prevent sheering. During this step, the angular position of the two aforementioned linkages is matched by adding another PI controller in series (Figure 6). The cascade of PI controllers outputs the desired speed of the EPW seat pan *y*_2_*(t)* to follow the angular position of the bed leg rest.

**Figure 7:**
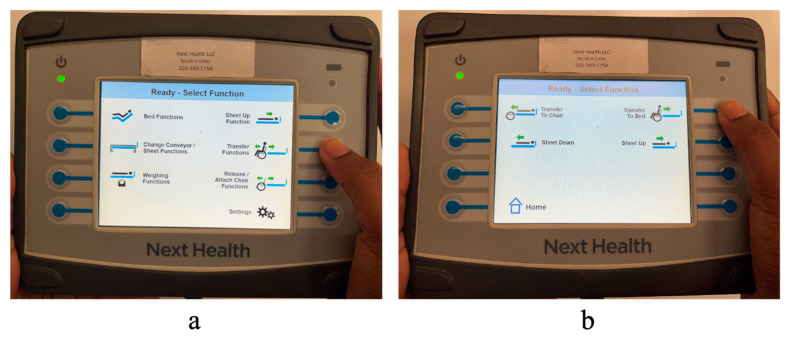
(**a**) Users interface to control PPTS main screen. (**b**) Transfer function screen.

The PPTS can be operated either using a hand-held primary user interface or a pendant controller. The primary user interface consists of a 7″ touchscreen with 8 soft keys and can be used to control all of the aforementioned functions of the PPTS (Figure 7). The pendant controller is smaller with 10 fixed most frequently-used functions, anticipated to be used by the person while in bed. The primary user interface and the pendant can be plugged into one of the four ports located on the bed. The main microcontroller communicates with these user interfaces (UI) via a 2-wire RS-485 using universal asynchronous receiver/transmitter (UART) protocol for serial communication. The main controller initiates all communications with UI by polling. When a user operates the UI, the UI sends data to the main controller indicating which system function is being requested. The main controller can then command corresponding motor controllers to accomplish that function using the aforementioned PI control.

### 2.2. Transfer Process

**Figure 8:**
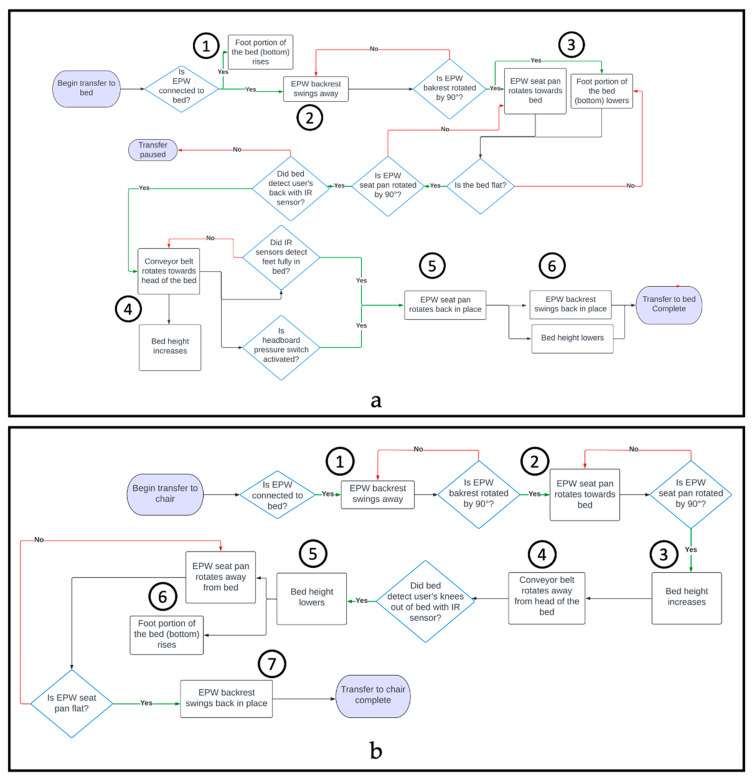
Flow Diagram. (**a**) EPW to bed transfer process. (**b**) Bed to EPW transfer process.

**Figure 9:**
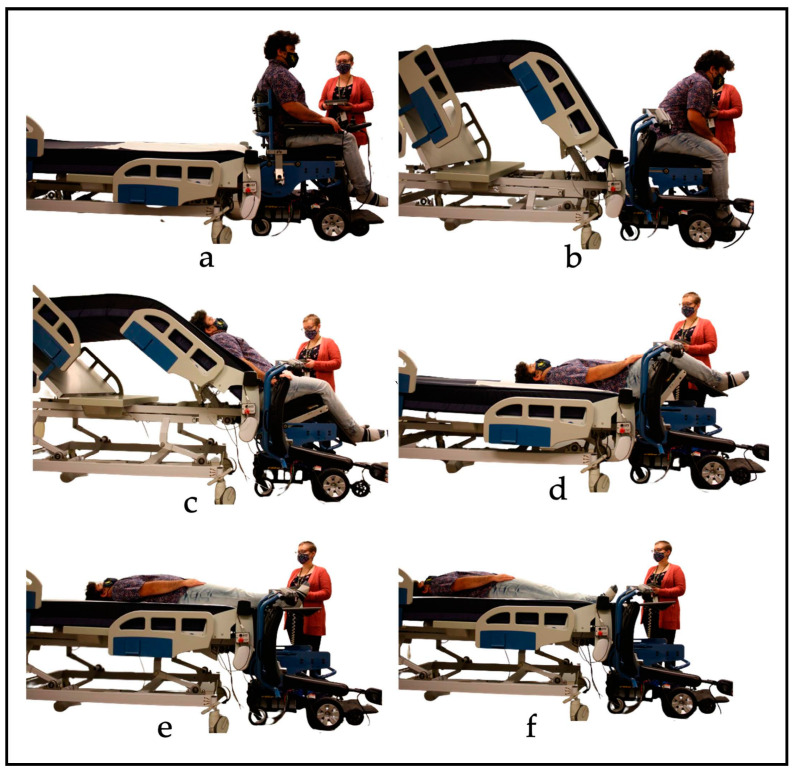
Transfer to bed process. (**a**) Initial position: Caregiver selects ’Transfer to Bed’ on controller. (**b**) EPW Backrest Swings Away. (**c**) EPW seat pan rotates towards the bed. (**d**) Bed lowers in sync with EPW seat pan. (**e**) Conveyor on the top of the bed pulls the user into the bed. (**f**) Bed height increases to smoothly transfer feet while conveyor continues to pull user into bed.

To begin the transfer, the EPW must be plugged into the bed to immobilize the EPW. This was accomplished via a cable extended from the bed and plugged into the charging port of the EPW, which activates the chair’s internal inhibit function and prevents movement. To transfer to/from the bed, the caregiver selects the “Transfer function” option on the primary user interface, as shown in Figure 7a. A second screen provides the options to “Transfer to Bed” or “Transfer to Chair” (Figure 7b). To start the transfer process, the caregiver must press and hold the desired button (‘Transfer to Bed’ or ‘Transfer to Chair’) until the process is completed. The caregiver can pause the transfer at any time by releasing the button. Figure 8a shows all the steps of the transfer in a flowchart. When the transfer begins, the EPW backrest swings away, as shown in Figure 9b, and the bed rises simultaneously with its leg rest to support the person’s back. The caregiver can assist the EPW user by supporting them as they lean forward while the backrest moves. Next, the EPW seat pan rotates towards the bed to rest the person’s back on the mattress (Figure 9c).

Once the person’s back is on the mattress, the EPW seat pan and bed leg rest move in sync until the person is lying flat on their back (supine position, Figure 9d). Such synchronization minimizes shear in the back and hips of the user. Next, the conveyor sheet starts to pull the user into the bed, and the bed height rises while the EPW seat pan continues to rotate so that the user’s feet are not dragged over the wheelchair (Figure 9e). Infrared sensors at the foot of the bed detect when the user’s feet have crossed the bottom edge or when the pressure bumper switch in the headboard detects that the head of the user has reached the top of the bed; the transfer is then complete. Position sensors in the bed and EPW linkages flag checkpoints when the actuators reach their desired position to continue to the next step in the transfer process, as shown in the flow charts.

To transfer back to the chair, the caregiver selects the transfer function option on the interface, then presses and holds “Transfer to chair”. Figure 8b shows the steps for the chair-to-bed transfer. Before transferring back into the chair, the PPTS alerts the caregiver to remove all blankets and confirm the transfer area is clear. The transfer process reverses itself, and the conveyor tightens and moves toward the foot of the mattress, pulling the user’s feet first towards the chair. Infrared sensors positioned at the end of the bed detect when the individual’s feet and then knees reach the chair. The bed begins to lower so that the person’s legs approach the chair in an appropriate orientation once the sensors detect the knees. The bed lifts up while the seat rotates to bring the person back into a seated position. Once the person is in a fully seated position, the backrest swings back into position. The caregiver may assist by leaning the wheelchair user forward, ensuring there is enough clearance for the backrest to lock into place. The EPW can then be unplugged from the bed to drive.

### 2.3. PPTS Kinematic Analysis

The kinematics of the PPTS system were verified using motion capture. Twenty infrared marker-based motion capture (MoCap) cameras from Vicon T40-S (Vicon Motion Systems, Culver City, LA, USA) [28] were used to track reflective markers placed on each linkage of the bed and EPW. Infrared marker-based MoCap systems have high precision and are accepted as the gold standard for motion analysis [29,30]. The Nexus software offered by Vicon was used to calibrate the cameras and record (x,y,z) positions of each marker (*f* = 100 Hz) while operating the PPTS to complete transfers between the bed and EPW. The position data from all markers were imported into MATLAB and analyzed to study angles (*θ*_1_ = bed headrest, *θ*_2_ = bed footrest, *θ*_3_ = angle to measure bed height, *θ*_4_ = EPW backrest, and *θ*_5_ = EPW seat rotation), changes in bed height, and conveyor displacement.

### 2.4. Focus Groups

#### 2.4.1. Participants

Focus groups were conducted to gather qualitative feedback about the PPTS. The study was approved by the Department of Veterans Affairs Pittsburgh Healthcare System (VAPHS) Institutional Review Board (IRB). Participants were recruited from local research registries at the Human Engineering Research Laboratories, through flyers distributed to clinics, consumer organizations, and at the National Veterans Wheelchair Games. Participants were screened for eligibility to participate in the study. The end-users were required to meet the following inclusion criteria: (1) 18 years of age or older, (2) were a US veteran, (3) use a mobility device (including manual wheelchairs and power wheelchairs), and (4) have the online capability to participate in a focus group (if attending virtually). Caregivers met the inclusion criteria of (1) 18 years of age or older, (2) have experience assisting a wheelchair user with transfers as a physician, nurse, physical therapist, formal caregiver (paid), or informal caregiver (family or friends), and (3) have the online capability to participate in a focus group (if attending virtually). The study recruited 36 participants, including 18 veterans using a mobility device and 18 caregivers experienced with assisting wheelchair users.

Participants took part in small focus groups, with each focus group consisting of one to six participants. Consent to participate was obtained from each participant. Demographic information was obtained from all participants. Focus groups followed a hybrid model where participants had the choice to either attend the focus group in person or remotely via Microsoft Teams. During the focus group, participants were given an overview of the system, followed by a prerecorded video demonstrating the functionality of the PPTS prototype (provided in Supplementary Materials S1). Participants were then asked to provide feedback based on a series of questions. Participants were asked about their overall impressions, advantages, and limitations of the system. Additionally, questions asked in the focus group included questions about the ease of use, safety, comfort, and demographic of people that would benefit from the PPTS. Participants were also provided an opportunity to share any other thoughts or ideas. Each focus group lasted less than an hour. Feedback from the participants was recorded and transcribed for analysis.

#### 2.4.2. Focus Group Data Analysis

Qualitative feedback from the participants was recorded, transcribed, and grouped based on distinct themes to reveal the advantages and limitations of the PPTS. Themes were coded using grounding theory. Participant responses to open-ended questions were first grouped based on mechanical, electronic, and software considerations and general concerns. Similar concerns from each of the groups were combined to form distinct themes. The analyzed data were reviewed by a multidisciplinary team, including assistive technology experts, rehabilitation professionals, engineers, and researchers, consistent with the Delphi process [31].

## 3. Results

### 3.1. PPTS System Verification

**Figure 10:**
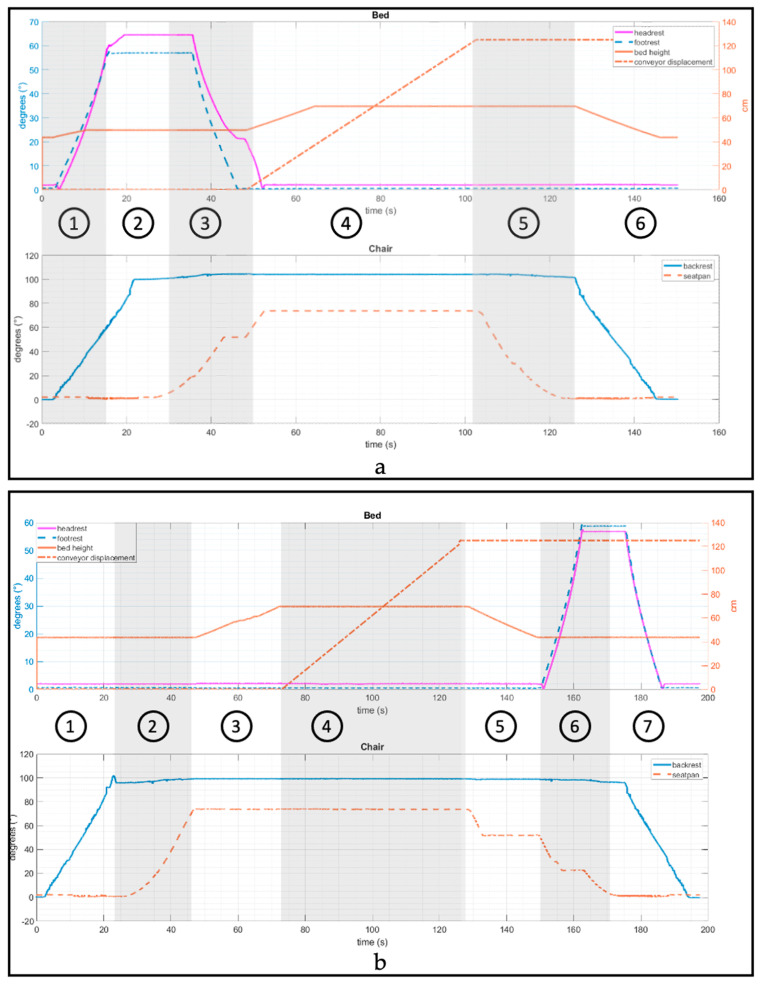
Kinematic Analysis of PPTS during (**a**) EPW to bed transfer and (**b**) Bed to EPW transfer.

Figure 10 shows the kinematics of the bed and EPW linkages during a transfer from EPW to bed. The graph is divided into shaded regions with circled numbers that correspond to the sequence of events established in the flow chart (refer to Figure 8). (1) As the transfer EPW-to-bed begins, the bed leg rest starts rotating at a constant average angular velocity ($\frac{d\theta_{2}}{dt}$ = 4.49 deg/s or 0.024Π rad/s) with a range of motion of 0–64°). (2) Next, the EPW backrest swings away from 0° to 100° with an average angular velocity of 5.16 deg/s or 0.028Π rad/s). The overshoot from the desired 90° to 100° is caused by the backrest load and joint play. (3) The bed leg rest lowers back to 0° (−5.40 deg/s or −0.03Π rad/s) while the EPW seat pan rotates towards the bed from 0° to 75° at the same angular speed. (4) Then, the conveyor moves the person into the bed with a linear velocity of 2.31 cm/s. The amount of time the conveyor moves varies by the height of the user. In addition, the bed height rises from 23 cm to 36 cm (velocity = 1.23 cm/s). (5) The EPW seat pan rotates back in place. (6) Last, the bed height lowers back to the minimum position, and the EPW backrest swings back in place. Similarly, Figure 10b shows the kinematics for a bed-to-EPW transfer. It is verified from the kinematic analysis that the bed and EPW follow the set and sequence of motions they were designed for.

**Figure 11:**
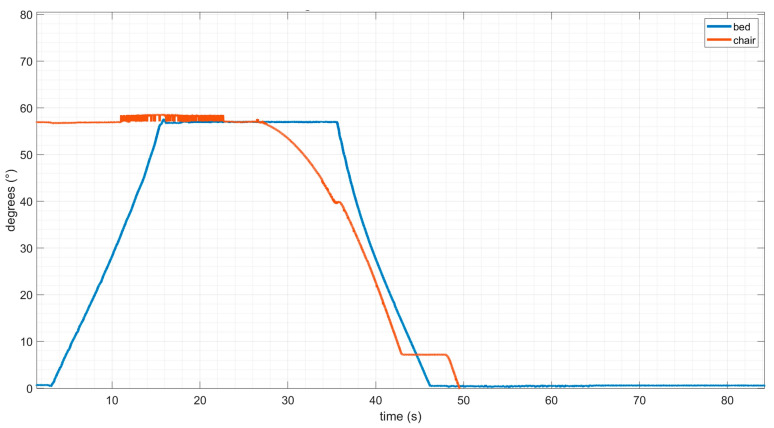
Orientation of seat pan controlled to match orientation of bed leg rest using PD control.

During step 3 of EPW-to-bed transfer, the bed leg rest and EPW seat pan angular speeds were synchronized using a PI controller. Figure 11 shows that there is a slight delay in the angular orientation of the bed leg-rest compared to the EPW seat-pan; however, it catches up to closely follow the similar angular orientation.

The validation process included an assessment of the equations pertaining to angles *θ*_1_–*θ*_5_, which were found to be in close agreement with the measured values. Based on the actuator stride of each actuator, the corresponding angles were calculated. Subsequently, a comparison was made between the calculated angles and measured angles, as well as between the calculated and measured bed height during the transfer process. The average errors between the theoretical values (derived from the equations) and the measured values were computed as follows. For the angle of the bed headrest (*θ*_1_), the average error (*σ*_1_) was determined to be 5.22°, corresponding to an error percentage of 4.42%. Similarly, the average errors for *θ*_2_ (bed footrest angle), *θ*_4_ (EPW backrest angle), and *θ*_5_ (EPW seat rotation angle) were found to be *σ*_2_ = 2° (error percentage = 8.3%), *σ*_4_ = 1.95° (error percentage = 1.22%), and *σ*_5_ = 4.27° (error percentage = 8.85%), respectively. Notably, the angle *θ*_3_ was employed in the calculation of the bed’s height. The discrepancy between the measured height and the calculated height was determined to be *σ*_3_ = 0.33 cm, equating to an error percentage of 0.65%.

### 3.2. Focus Group Participants and Demographics

Demographic information (N = 36) of participants, including 18 veterans with disabilities using a mobility device and 18 caregivers, are summarized in Table 1. The 18 mobility device users that participated in the focus groups included 15 male and 3 female participants. The 18 caregivers/rehabilitation professionals that participated in the focus groups included 15 females and 3 males.

### 3.3. Focus Group Results

#### 3.3.1. Overall Impressions

The majority of participants reported positive overall impressions of the PPTS. Participants perceived the system to be innovative and useful, which could reduce the likelihood of injury and human error. Participants reported that the PPTS seemed easy to use, would reduce caregiver effort while performing transfers, and could be beneficial in clinical settings. People with experience in using mechanical transfer lifts reported that the PPTS could be a significant improvement over mechanical lifts. Table 2 outlines the overall impressions of the participants in descending order of the number of times reported, i.e., most reported at the top and least reported at the bottom of the table. A participant expressed that “I like the idea of it making people more independent”. Other responses reported that the PPTS would facilitate an increased feeling of wellness and quick transfers. Conversely, only 1 of the 36 participants described the PPTS to be cumbersome and expensive in their overall perception.

#### 3.3.2. Best Features

Most participants reported that the functionality of the PPTS to reposition the person towards or away from the bed (longitudinal repositioning) is the best feature of the bed, as shown in Table 3. The conveyor belt on the hospital bed eliminates the need for the caregiver to physically pull their care recipient to reposition them. This ensures that if the care recipient slides down from the pillow area, the conveyor system can be activated to position them back. Participants reported that the PPTS seemed easy to use and a more secure way of transferring between a wheelchair and a bed. Participants expressed that the PPTS would make transfers easy and consistent. Since the PPTS makes transfers highly automated, it eliminates the inconsistencies in assisted transfers by different caregivers at different times. One participant expressed, “The transfers will feel and function the exact same way each time. I can get used to it”. Some participants reported that they liked the backrest design. The addition of actuators to the Group-2 wheelchair enables the custom backrest to swing away so the user can be transferred into the bed. Since the PPTS can be operated using a hand-held user interface, participants expressed that the PPTS seemed easy to use and would require minimal caregiver effort.

#### 3.3.3. People That Can Be Served by PPTS

Participants reported that people with several demographics can benefit from the PPTS. The following population of care recipients would benefit according to responses received in the focus group: (1) anyone that needs to transfer; (2) people with spinal cord injury; (3) people with critically deteriorating health; (4) overweight, obese, and tall people; (5) older adults; (6) people with low dexterity; (7) people with late stages of Parkinson’s disease; (8) people with muscular dystrophy; (9) patients admitted to in-patient hospitals or rehabilitation facility. Additionally, caregivers that would most benefit from the technology include (1) older caregivers, (2) busy caregivers with time constraints, and (3) patients and nurses in ICUs and hospitals. Based on the responses received in the focus groups, the PPTS would benefit both the caregiver and the care recipient in a variety of settings, including home, clinical, rehabilitation facilities, and assisted living settings.

#### 3.3.4. Usability and Automation

The majority of participants (N = 35) reported that the PPTS would make wheelchair-to-bed transfers safer and easier. Additionally, all 18 mobility device users in the study expressed that they would feel comfortable with fully autonomous, computer-controlled transfers. One participant expressed that as long as an emergency stop was built, similar to the one included in the PPTS, autonomous transfer would be preferable. Fifteen of the eighteen mobility device users would feel confident in using the PPTS independently, while three participants preferred to be under the supervision of a caregiver. While the current prototype of the PPTS is expected to be controlled by the caregiver, future design of the PPTS may provide more functionality to aid in independent transfers.

#### 3.3.5. Limitations of PPTS

Participants were asked to discuss the least desirable features, missing features, suggested changes, and stigmas associated with the device. Responses received in the focus group were grouped based on similar themes to report the limitations of the PPTS, as shown in Table 4.

The majority of participants mentioned the limitations related to the Group-2 wheelchair. The current wheelchair prototype lacks power seat functions for pressure relief. While the seat cushions can be customized depending on user needs, it does not have tilt and recline functions. In addition, the Group-2 wheelchairs do not support an in-built toilet. However, it should be noted that such features are not commonly available on any Group-2 wheelchairs. Power seat functions are featured on Group-3 wheelchairs, while a built-in toilet is only available with a commode-specific manual wheelchair.

Participants expressed the desire to have the ability to perform transfers independently without the assistance of the caregiver. While transferring the care recipient from the bed to the wheelchair and vice versa, the backrest of the wheelchair swings away. If the care recipient lacks trunk control, caregiver assistance may be required to support the care recipient at this point as they lean forward. The current prototype of the PPTS does not feature a mechanism for trunk support, which was a reported limitation.

While transferring from the bed to the wheelchair, the care recipient has to laterally align the user in the center of the bed so the conveyor can transfer them onto the wheelchair. Additionally, minor assistance from the caregiver may be required in lifting the user’s feet while they are being transferred from the conveyor to the footrest of the chair. This warrants a specific leg orientation and minor assistance by lifting legs, which was identified as a limitation. Other infrequent limitations expressed by participants include the need for a large space for the bed and chair, slow speed, and concerns regarding high cost and insurance coverage. Additionally, the current prototype does not assist with rolling over or medially sliding the care recipient.

Some participants mentioned the limitation of the primary user interface to control the PPTS. The need to integrate a modern touchscreen with fewer tactile buttons was expressed in serving people with lower arm strength and dexterity. Additionally, the ability to control the PPTS via mobile phone application and other modalities such as speech was recommended.

The PPTS currently supports users that weigh under 350 lbs. Participants pointed out that the weight restriction would limit access to the bariatric population. The design is suitable for heavier people but would need to be compatible with a bariatric wheelchair.

## 4. Discussion

Technologies to assist with mobility and transfers are among the most important tools that can be used to promote wellness, independence, community participation, and quality of life [32]. The development of technological solutions for mobility and transfers is critical given the aging population of the United States, the increased cost of care, and the shortage of professional caregivers [33,34].

The focus groups revealed the advantages and limitations of the PPTS. A majority of participants reported that the PPTS would aid in increased mobility and independence. The PPTS eliminates the need for lifting the care recipient, either manually or using a mechanical lift. This is expected to result in a lower chance of injury for the caregiver and their care recipient. Additionally, transfers with the PPTS are consistent, which reduces the variability due to human error involved with manual transfers.

A recent study reported that arm forces and spine compression exceed safe limits when repositioning people in bed [35,36]. The PPTS presents a significant advantage in this regard by eliminating the need for manually repositioning the care recipient between the head and foot of the bed.

Participants in the study reported that the PPTS would be beneficial to a wide variety of demographics. Caregivers assisting with transfers, especially older caregivers and nurses that assist with transfers with a high frequency, would benefit from the PPTS the most. Additionally, wheelchair users, older adults, and people with critical and deteriorating health conditions are expected to benefit from the PPTS. The focus groups revealed that the bariatric population and their caregivers could benefit from a transfer technology such as the PPTS. The applications of the PPTS are appropriate in a home setting as well as a clinical setting. In addition, nursing homes, assisted living facilities and physical rehabilitation facilities may benefit from reliable and safer transfers enabled by the PPTS.

All the mobility device users in the study reported a preference for a fully automated or computer-controlled autonomous transfer system. In addition, a large majority (97%) reported that they would feel comfortable using the PPTS independently if such provisions were designed in future prototypes of the PPTS. This is consistent with the previous voice of consumer research, which highlighted autonomous and intelligent assistive technologies to be a high priority for mobility device users [2].

Several areas for further investigation were revealed in the focus groups. The PPTS does not accommodate the needs of Medicare Group-3 or Group-4 wheelchair users that require custom backrests, postural supports, custom-molded seats, or bariatric patients. However, the current prototype of the PPTS was developed to serve Group 2 EPW users, which includes the largest population of United States EPW users. The focus group population was mostly users of Group 3 and 4 EPW, hence not the target population for this device. However, they provided useful information for the current device and future directions. Additionally, the end-user needs to be centrally aligned in the bed to have the correct leg orientation while transferring from the bed to the wheelchair. Currently, there is no provision to roll over the user or laterally slide them without human assistance. This may require a complete redesign with additional complexity. The caregiver may need to assist the user with low trunk control while leaning forward while the backrest swings away for the transfer. While these limitations restrict independent use by wheelchair users, they require minimal assistance from a caregiver.

The presented kinematic analyses will aid in the customization of the PPTS based on the specific user’s needs in the future. It also provides a pathway to further refine mechanisms and interface different wheelchairs (such as Group-3) with the PPTS.

Another limitation reported was that the current prototype of the PPTS is not suitable for the bariatric population due to weight restrictions. The Group-2 EPW was the limiting factor in this regard since it is rated for a maximum weight capacity of 159 kg (350 pounds). However, the bed can support up to 272 kg (600 pounds), which may make it feasible to extend the PPTS to be interfaced with a bariatric EPW in the future.

Design considerations for the future development of the PPTS include creating a mechanism for stability while leaning, adaptive transfer speed control based on user comfort, and a mechanism to enable rolling over or sliding medially. Additionally, the PPTS may benefit from a user-friendly touchscreen controller and other modalities of instruction, including voice commands and mobile phone applications, to better serve people with low hand dexterity (e.g., end-users versus caregivers). However, the PPTS is currently designed to be operated by caregivers. The addition of these modalities could be implemented for independent use in the future.

Feedback from stakeholders is a critical aspect of the participatory action design and engineering process. This was facilitated via focus groups with wheelchair users and experienced caregivers to inform design modifications as a part of the iterative design process. Focus groups served as a preliminary assessment of the prototype, which will aid in the future refinement of the prototype to conduct actual subject-testing of transfers with wheelchair users and their caregivers.

Future work may focus on integrating a commode/shower wheelchair with the existing bed. Caregivers could swap wheelchairs while the user is in bed and transfer them into the commode/shower chair when needed. The user can then be transferred back into the bed to be transferred into the EPW, mitigating the need for transfers between different wheelchairs.

Some general limitations of the PPTS include the cost to the user or family or concerns about insurance coverage, space requirements, and complex maintenance. Using the PPTS may require training and user compliance. These limitations are similar to other commercially available transfer technologies.

## 5. Limitations

This study is limited to the perceptions and opinions of mostly Group 3 and 4 EPW users and their caregivers. Future studies, including qualitative usability analysis and biomechanical evaluations, may further reveal areas of improvement in the PPTS. The study is limited to the perceptions of participants based on an online presentation and video demonstration of the system. A follow-up study involving caregivers and wheelchair users operating the PPTS for transfers needs to be conducted to test the usability of the system.

## 6. Conclusions

The benefits of the PPTS may outweigh its limitations, making it a promising solution for safer transfers. Overall, participants perceived the PPTS as a safer and more reliable solution for assisted transfers. Future work will focus on making improvements to the current prototype. Future design considerations include the addition of a mechanism for trunk support, customizable transfer speed, and a user-friendly touchscreen interface. Improvements to the chair aesthetics will be made as suggested by participants in the focus group.

## Figures and Tables

**Table 1:** Demographic information.

Variable	Mobility Device User	Caregivers/Rehabilitation Professionals
Gender	Female: 3 Male: 15	Female = 15 Male = 3
Age (years)	58.33 ± 10.72 (Range = 30–76)	42.6 ± 16.3 (Range = 25–74)
Height (m)	1.75 ± 0.064 (Range = 1.60–1.84)	1.67 ± 0.12 (Range = 1.5–1.88)
Weight (Kg)	90.10 ± 26.35 (Range= 49.50–164.70)	79.57 ± 18.44 (Range = 44.1–112.5)
Ethnicity	Caucasian = 13 African American = 3 Mixed = 1 Caribbean = 1	Caucasian = 11 African American = 2 Asian = 2 Hispanic = 1 Undisclosed = 2
Education Level	Bachelor Degree = 7 Master Degree = 6 High School Diploma or GED = 3 Associate Degree = 1 Vocational/Technical School = 1	
Occupation Level or Type of Caregiver	Retired = 10 Work part-time = 3 Work full-time = 2 Unemployed = 2 Volunteer = 1	Professional caregivers = 11 Physical therapists = 7 Personal care attendant = 2 Occupational therapist = 1 Nurse = 1 Informal Caregivers = 7
Mobility Device or Experience with mobility devices	Power Wheelchair = 11 Manual Wheelchair = 6 Scooter = 1	Manual wheelchairs = 17 Powered wheelchairs= 12 Scooter= 6 Recreational (handcycle, racing, quad rugby chairs) = 5
Disability or Disability demographic experience	SCI = 11 Neuromuscular diseases = 5 Cognitive impairment = 2	Neuromuscular Diseases (e.g., multiple sclerosis; muscular dystrophy, ALS) = 12 Spinal Cord Injury = 9 Cerebrovascular Disease (Stroke) = 8 Cardiovascular Disease = 7 Congenital impairment (e.g., cerebral palsy, spinal bifida) = 7 Cognitive impairment (e.g., traumatic brain injury, Alzheimer’s, and dementia) = 6 Orthopedic impairments = 5 Cardiopulmonary Disease = 4 Osteoarthritis = 3 Rheumatoid arthritis = 3
Number of hours assisting mobility device user (per week)		1–8 h = 3 8–16 h = 1 16–24 h = 2 24–32 h = 1 32–40 h = 6 40+ h = 5
Age of people providing care to		Children (<16 years) = 7 Adults (17–65 years) = 15 Seniors (65+ years) = 12

**Table 2:** Overall impressions of the PPTS as reported by participants, including mobility device users and caregivers.

Overall Impressions in Descending Order of Number of Times Reported	# of Participants
Great/Impressive/Innovative	17
Reduces the chance of injury and human error	8
Easy and Safe	4
Better than mechanical lifts	3
Reduces caregiver effort	3
Helpful in clinical settings	2
Cumbersome/expensive	1

**Table 3:** Key features of the PPTS as reported by participants.

Key Features of the PPTS in Descending Order of Number of Times Reported	# of Participants
Person repositioning	12
Transfers and consistency	6
Secure and easy	6
Low effort and human-interaction	2
Easy controls	2
EPW backrest design	2

**Table 4:** Limitations of the PPTS identified by participants.

Limitations of the PPTS in Descending Order of Number of Times Reported	# of Participants
Chair Limitations	19
Lack of independent transfers	10
Leg Orientation to transfer to the chair	9
Lacks trunk support	9
Cost/Insurance	5
Slow speed	5
Space constraints	5
Weight limitation	4
Limitations with user interface	4
Lacks lateral repositioning	4
Social stigmas	3
After-market modifications	3
Lacks support for prone transfers	3
Complex/hard maintenance	3
Needs additional fault detection	2
Chair looks institutional	2
Training requirement	2
Conveyer impediment to comfort	1
Patient compliance required	1

## Data Availability

To ensure the privacy and confidentiality of the human subjects involved in this project, all information and data are treated as confidential. Stringent privacy standards have been implemented to safeguard the personal and sensitive information of the participants. As a result, the data cannot be shared with external parties. The commitment to maintaining confidentiality is vital in upholding the ethical principles of the research and respecting the privacy rights of the individuals involved.

## Supplementary Materials

The following supporting information can be downloaded at: https://www.mdpi.com/article/10.3390/s23125540/s1, Presentation S1: Development and Evaluation of Powered Personal Transfer System Focus Group.
