# Supplementary material for: Participatory Action Design and Engineering of Powered Personal Transfer System for Wheelchair Users: Initial Design and Assessment
Source: Sensors (Basel). 2023 Jun 13;23(12):5540. doi: 10.3390/s23125540 (PMC10303711; doi:10.3390/s23125540)
Supplement: Supplementary file 1 [file sensors-23-05540-s001.zip › sensors-2350108-supplementary.pptx]

## Slide 1
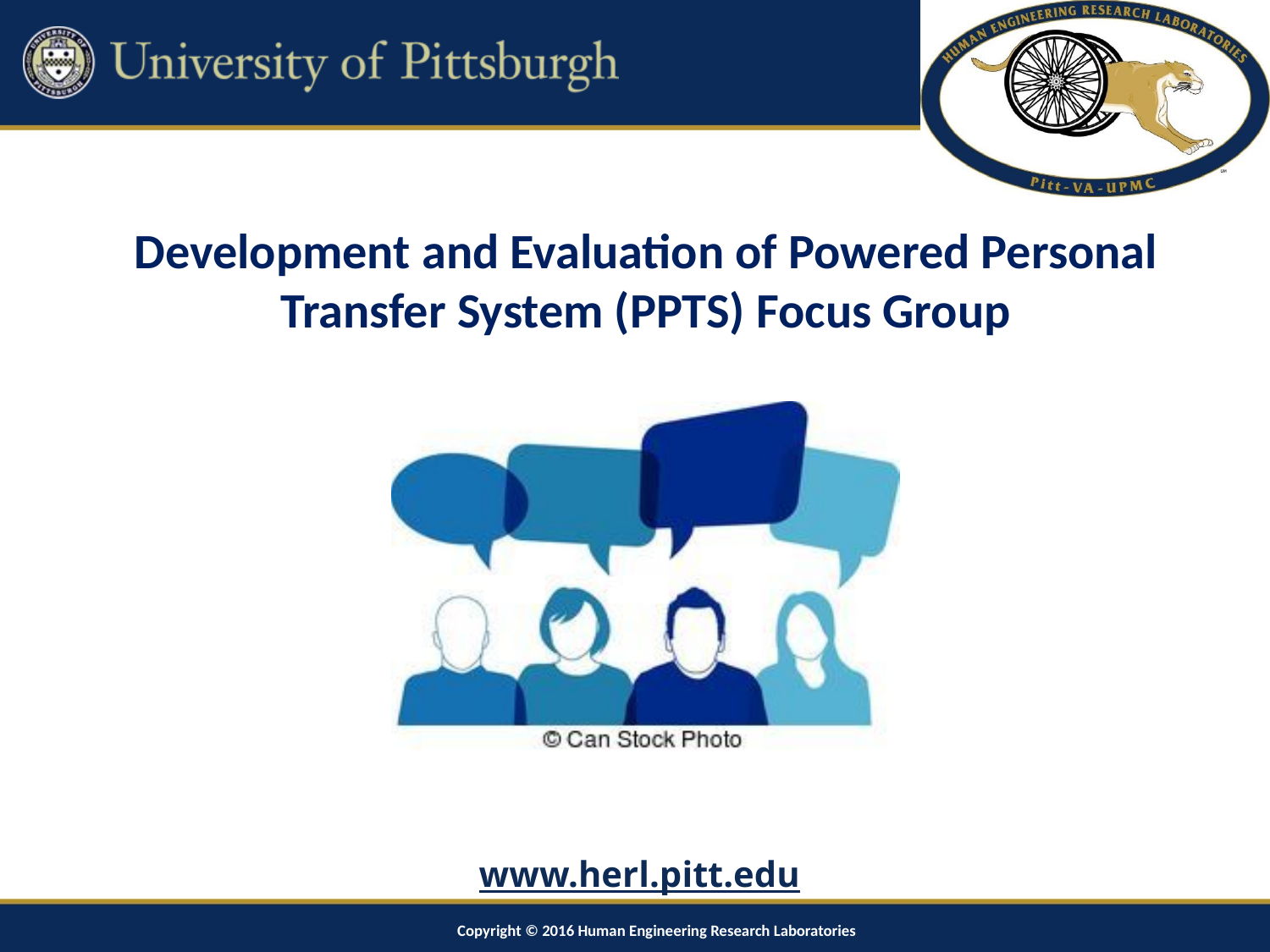

Development and Evaluation of Powered Personal Transfer System (PPTS) Focus Group
 www.herl.pitt.edu
Copyright © 2016 Human Engineering Research Laboratories

## Slide 2
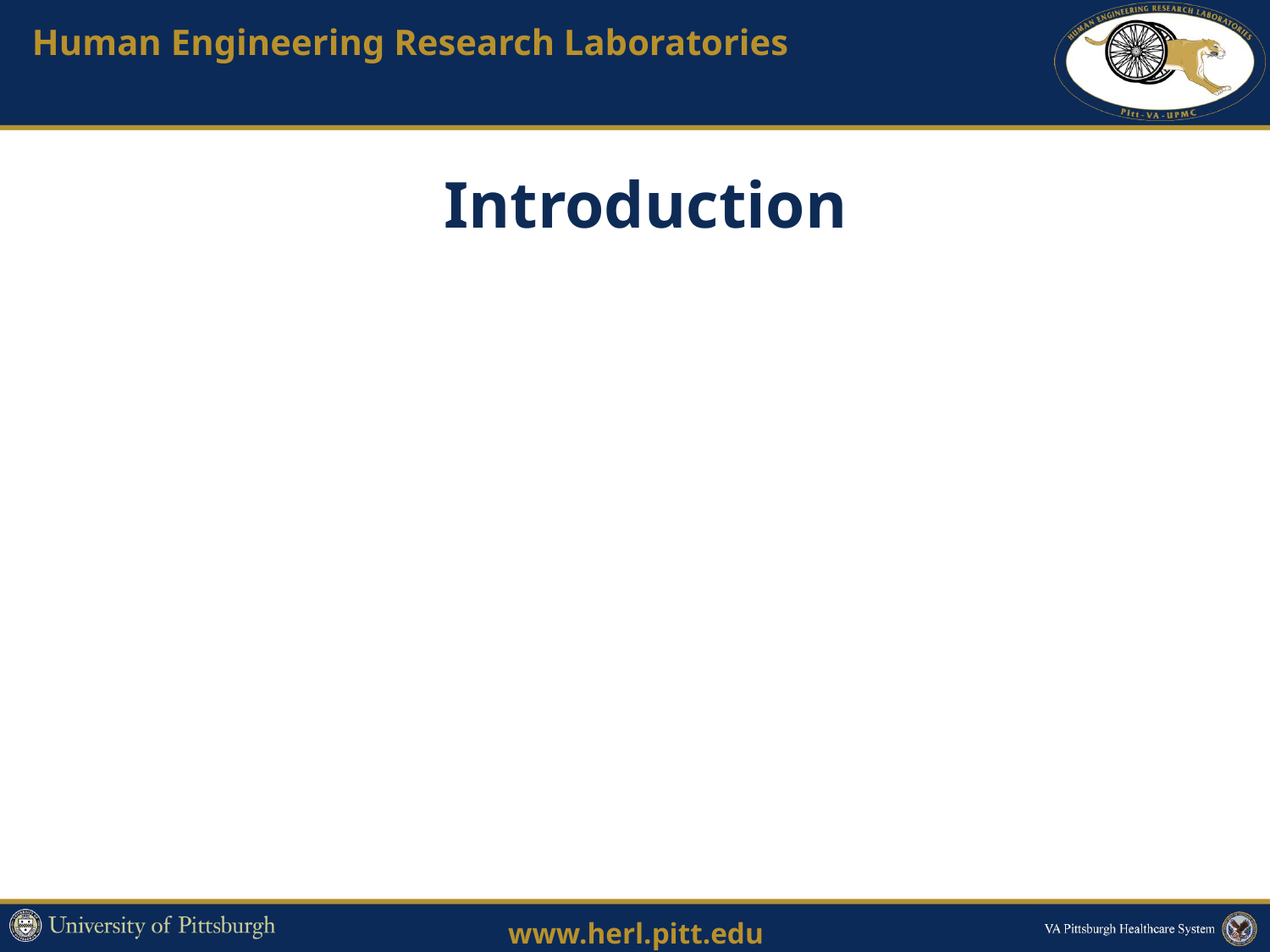

Human Engineering Research Laboratories
Introduction
 www.herl.pitt.edu

## Slide 3
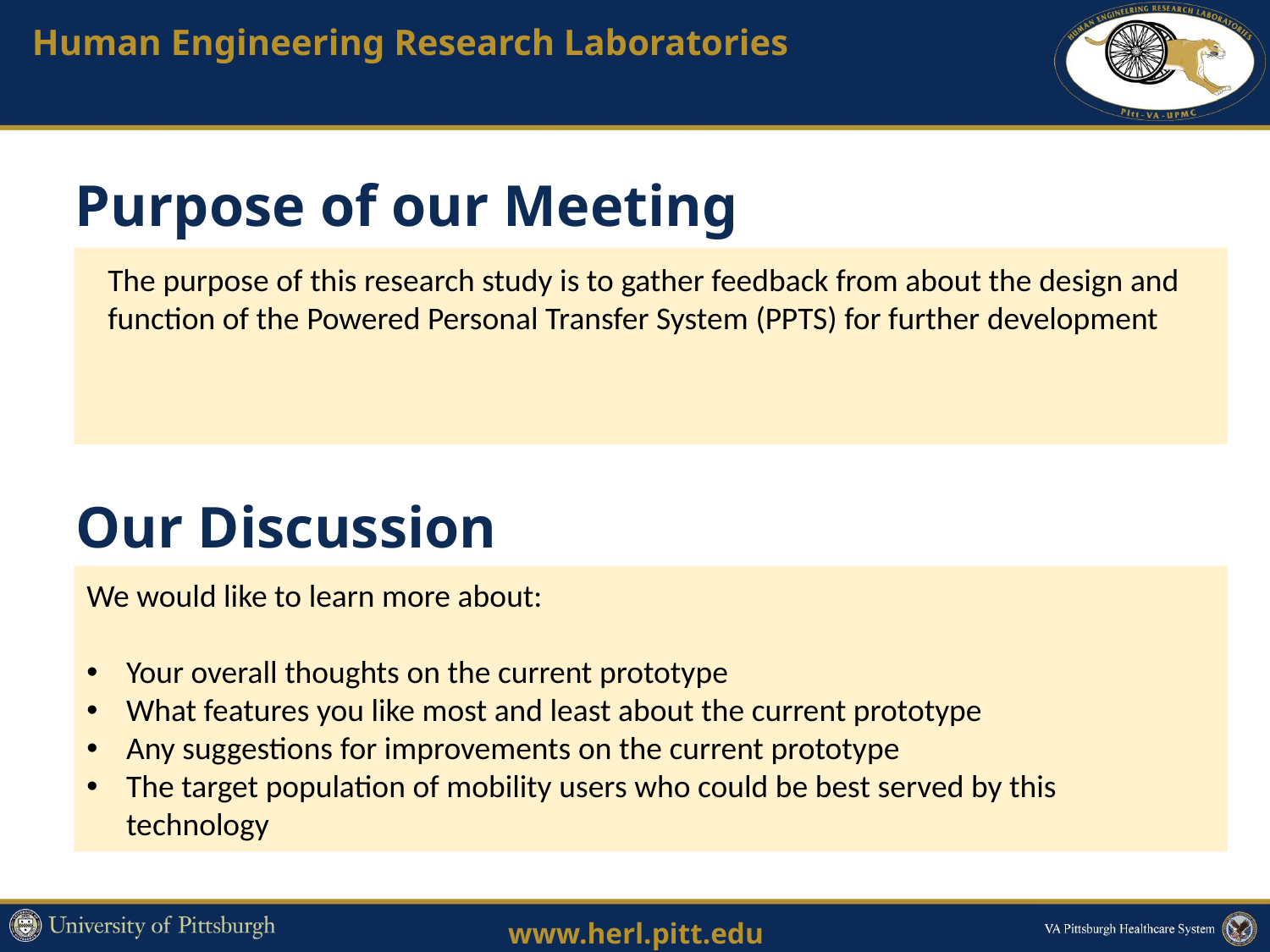

Human Engineering Research Laboratories
Purpose of our Meeting
The purpose of this research study is to gather feedback from about the design and function of the Powered Personal Transfer System (PPTS) for further development
Our Discussion
We would like to learn more about:
Your overall thoughts on the current prototype
What features you like most and least about the current prototype
Any suggestions for improvements on the current prototype
The target population of mobility users who could be best served by this technology
 www.herl.pitt.edu

## Slide 4
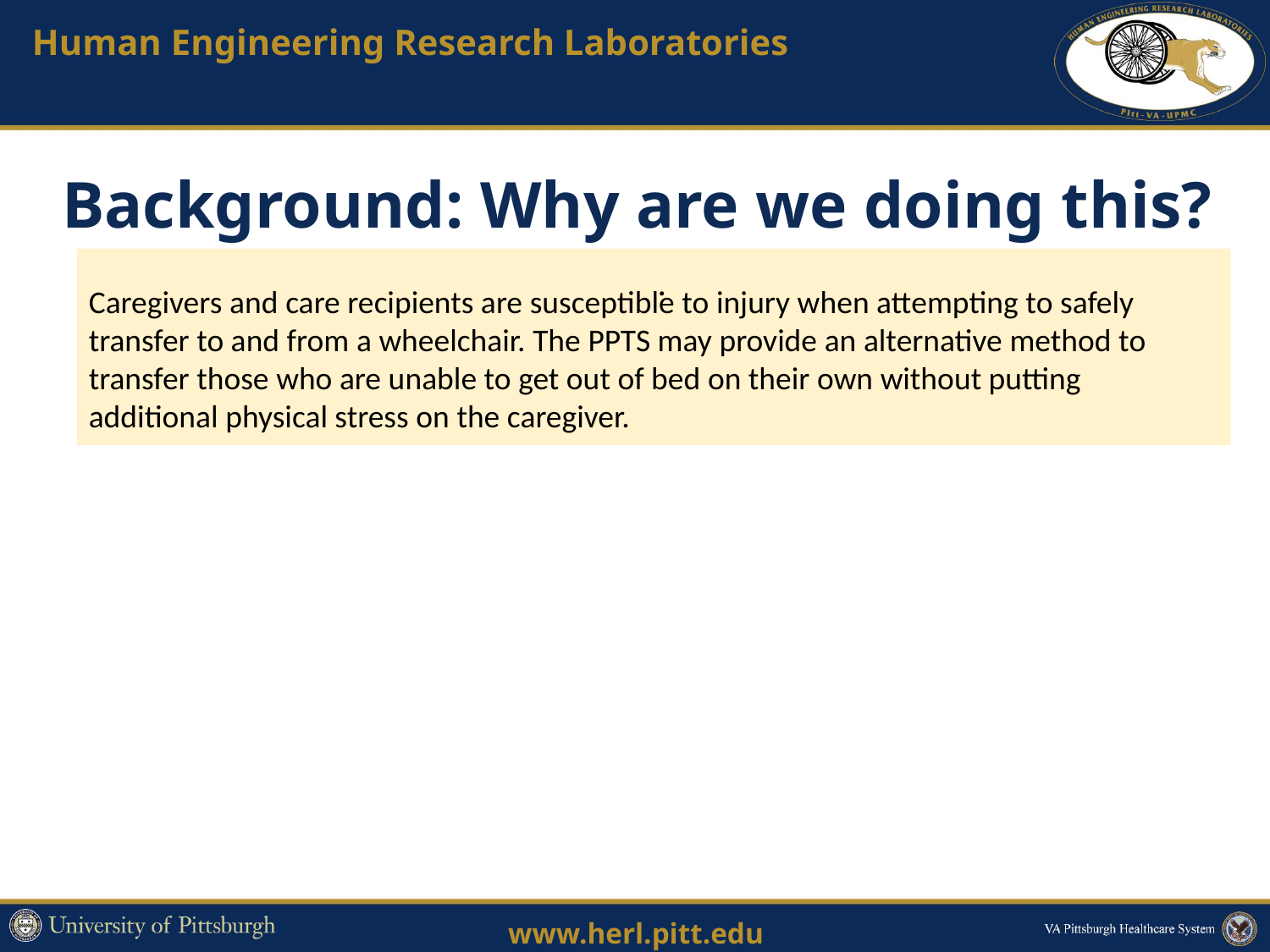

Human Engineering Research Laboratories
Background: Why are we doing this?
Caregivers and care recipients are susceptible to injury when attempting to safely transfer to and from a wheelchair. The PPTS may provide an alternative method to transfer those who are unable to get out of bed on their own without putting additional physical stress on the caregiver.
.
 www.herl.pitt.edu

## Slide 5
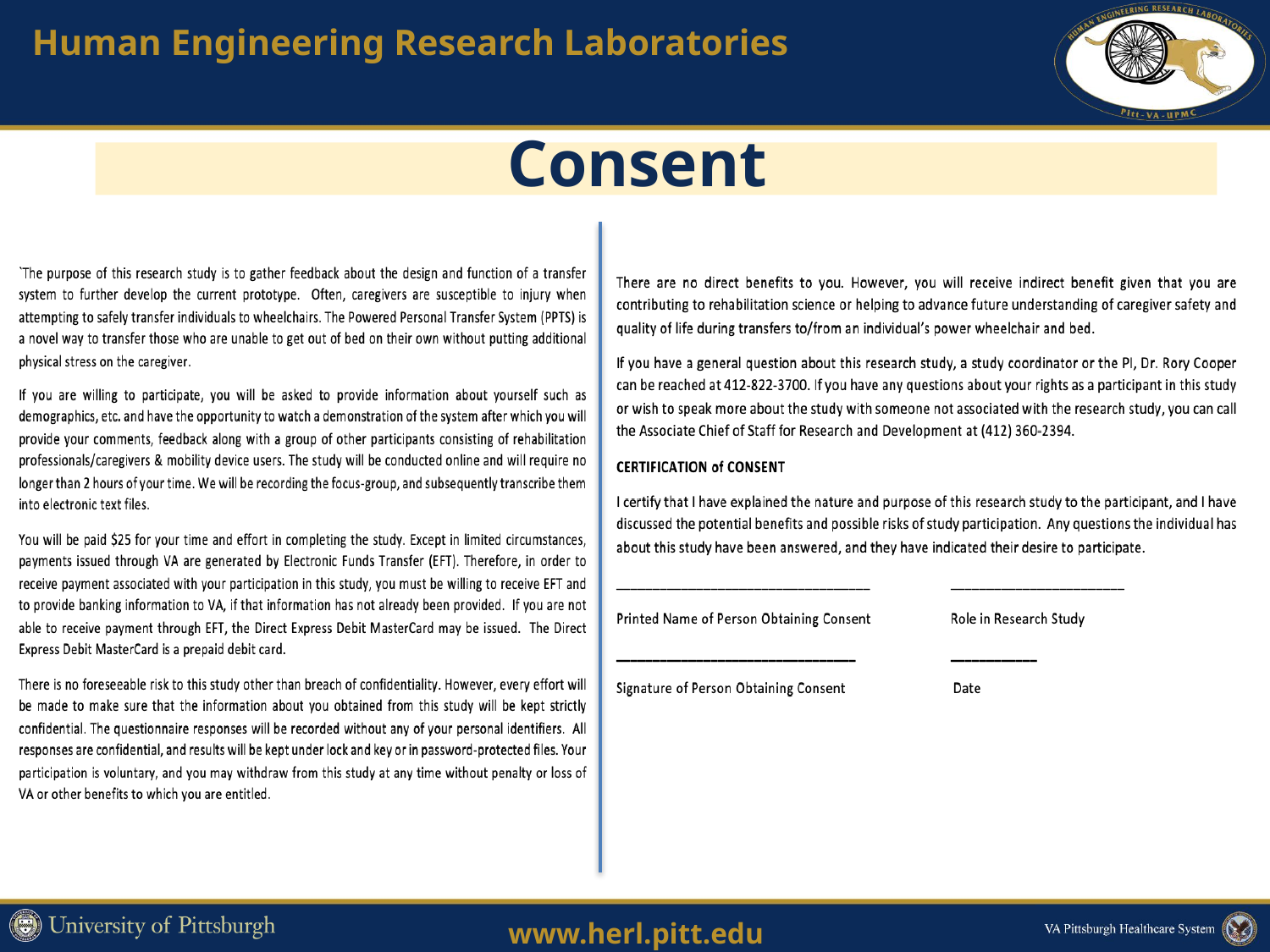

Human Engineering Research Laboratories
Consent
 www.herl.pitt.edu

## Slide 6
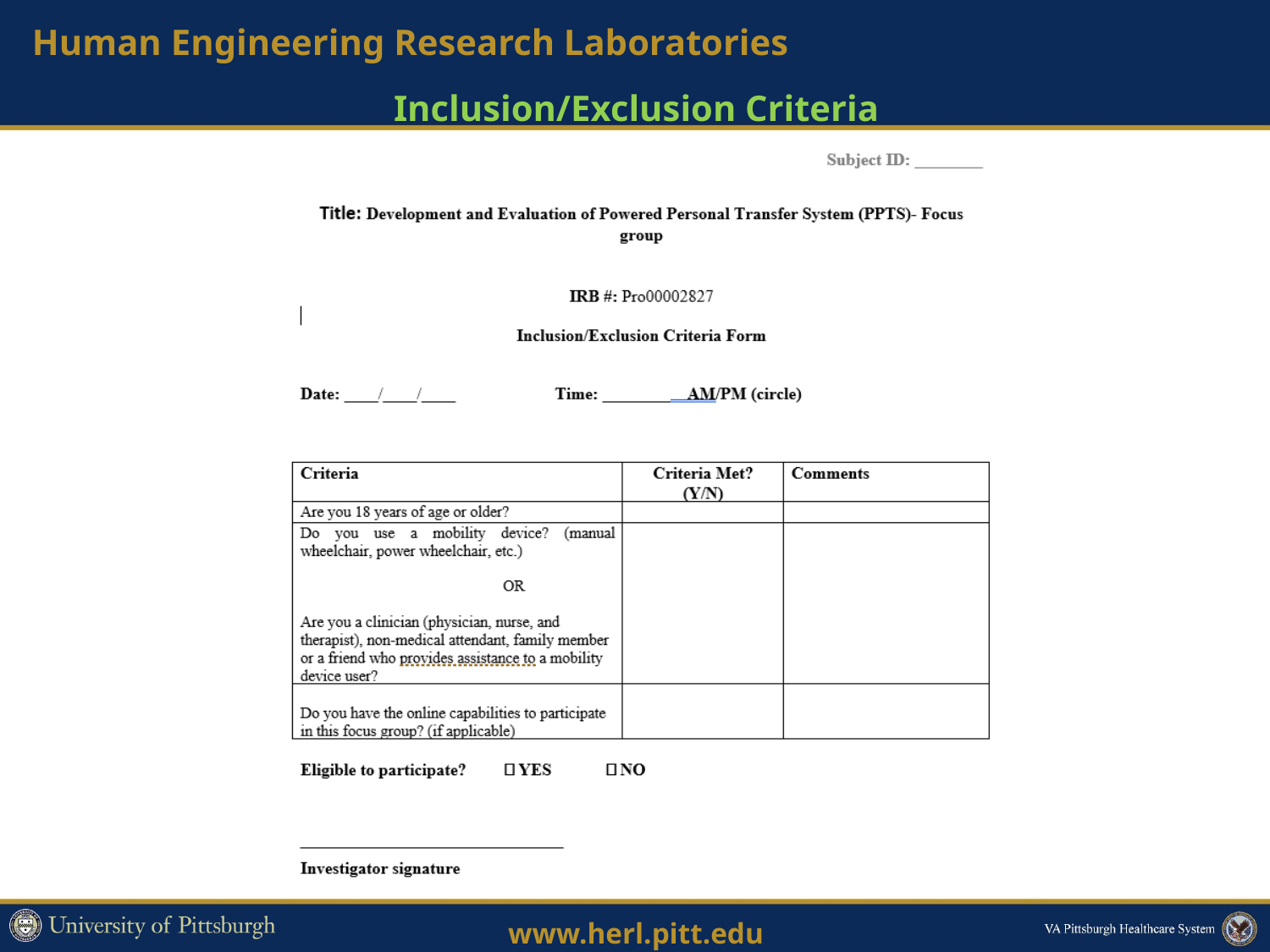

Human Engineering Research Laboratories
Inclusion/Exclusion Criteria
 www.herl.pitt.edu

## Slide 7
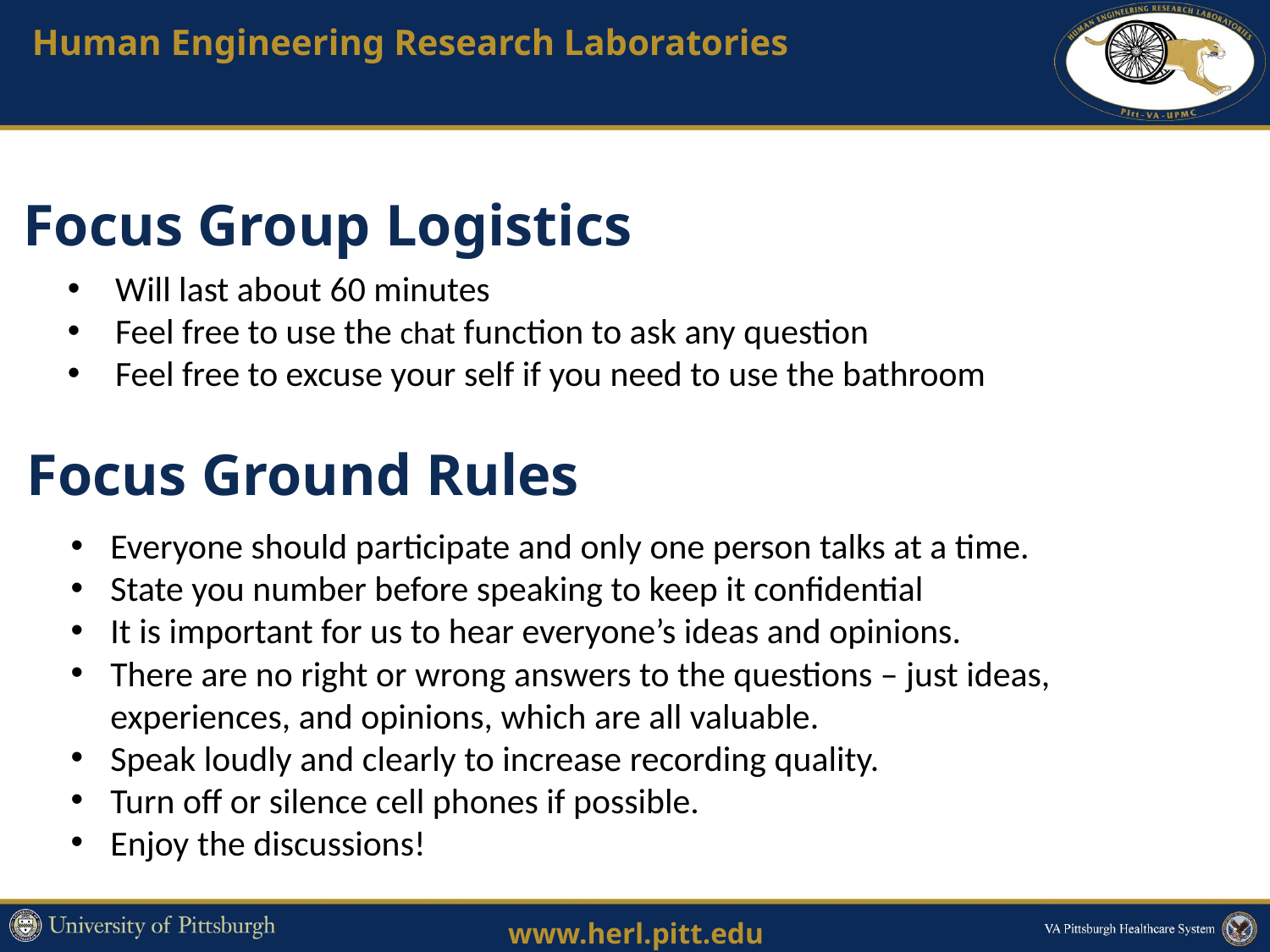

Human Engineering Research Laboratories
Focus Group Logistics
Will last about 60 minutes
Feel free to use the chat function to ask any question
Feel free to excuse your self if you need to use the bathroom
Focus Ground Rules
Everyone should participate and only one person talks at a time.
State you number before speaking to keep it confidential
It is important for us to hear everyone’s ideas and opinions.
There are no right or wrong answers to the questions – just ideas, experiences, and opinions, which are all valuable.
Speak loudly and clearly to increase recording quality.
Turn off or silence cell phones if possible.
Enjoy the discussions!
 www.herl.pitt.edu

## Slide 8
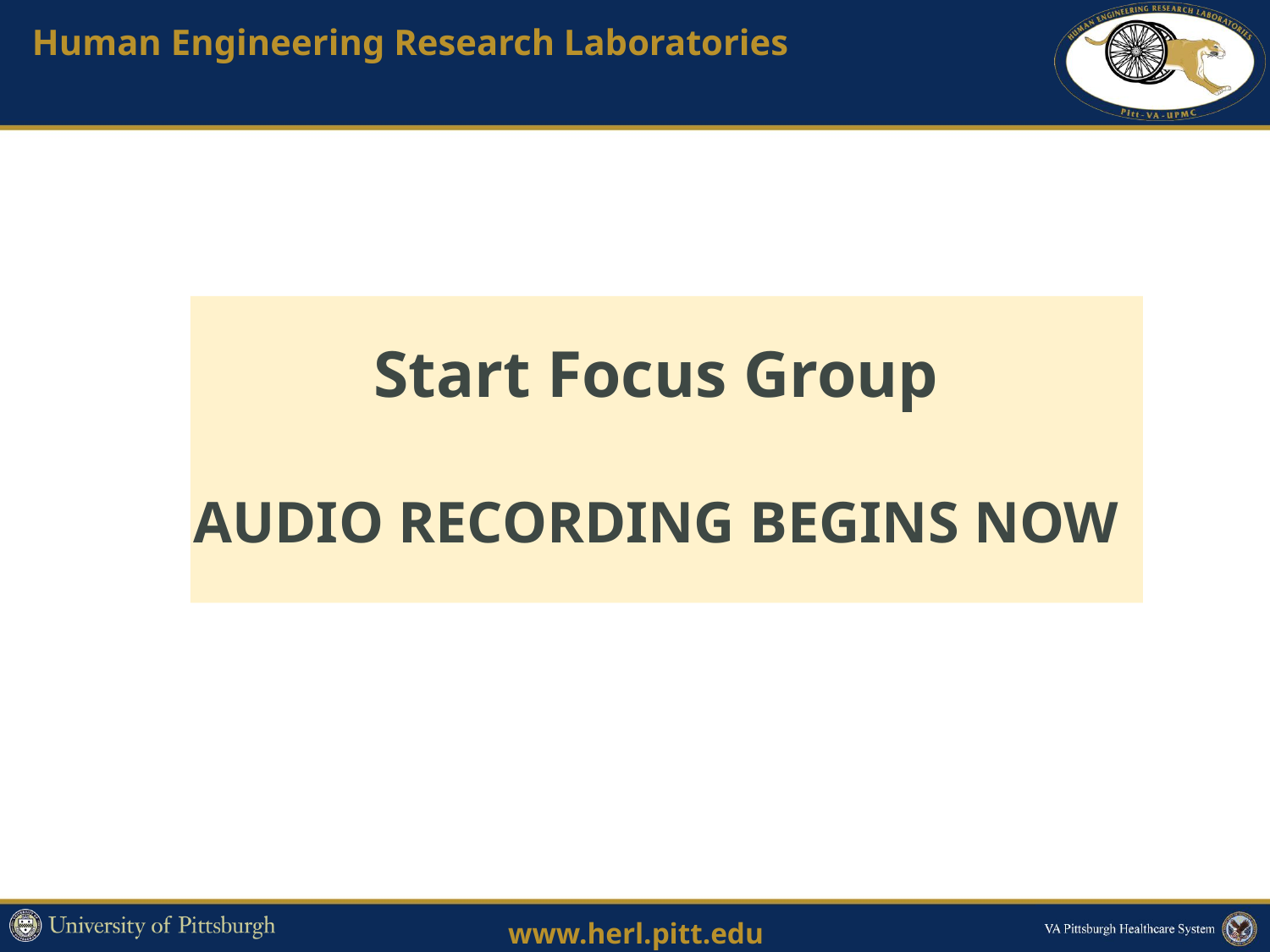

Human Engineering Research Laboratories
Start Focus Group
AUDIO RECORDING BEGINS NOW
 www.herl.pitt.edu

## Slide 9
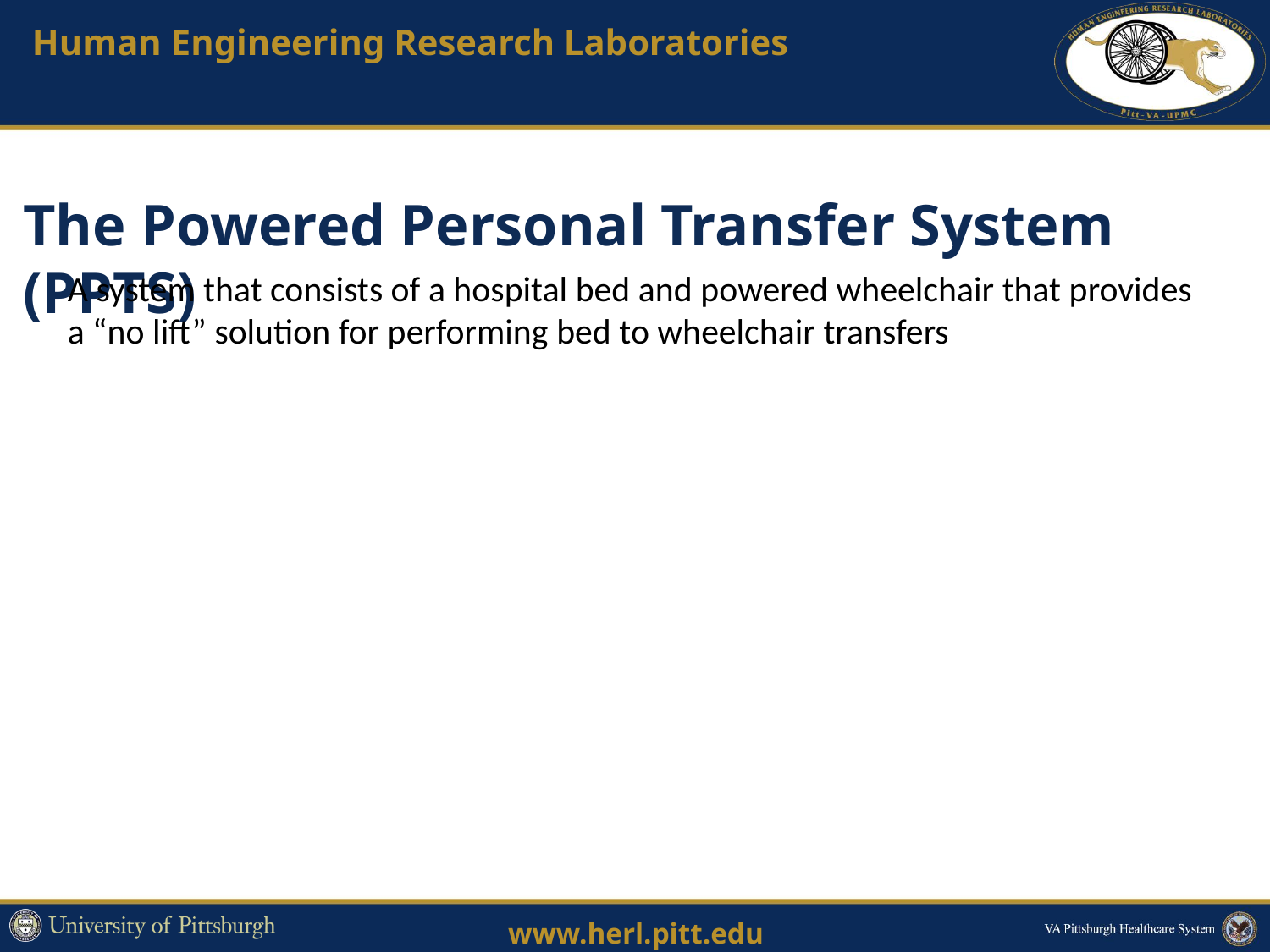

Human Engineering Research Laboratories
The Powered Personal Transfer System (PPTS)
A system that consists of a hospital bed and powered wheelchair that provides a “no lift” solution for performing bed to wheelchair transfers
 www.herl.pitt.edu

## Slide 10
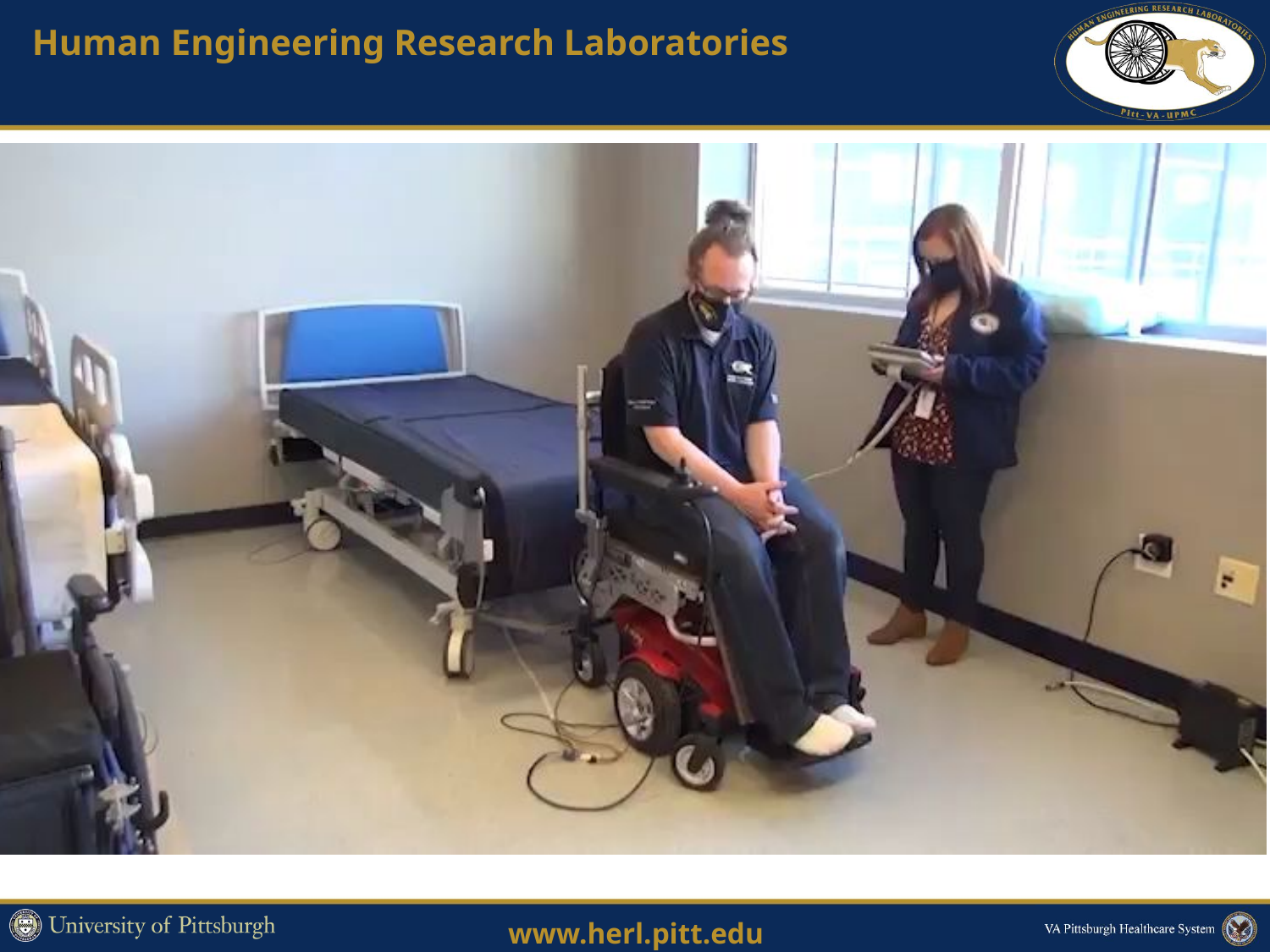

Human Engineering Research Laboratories
 www.herl.pitt.edu

## Slide 11
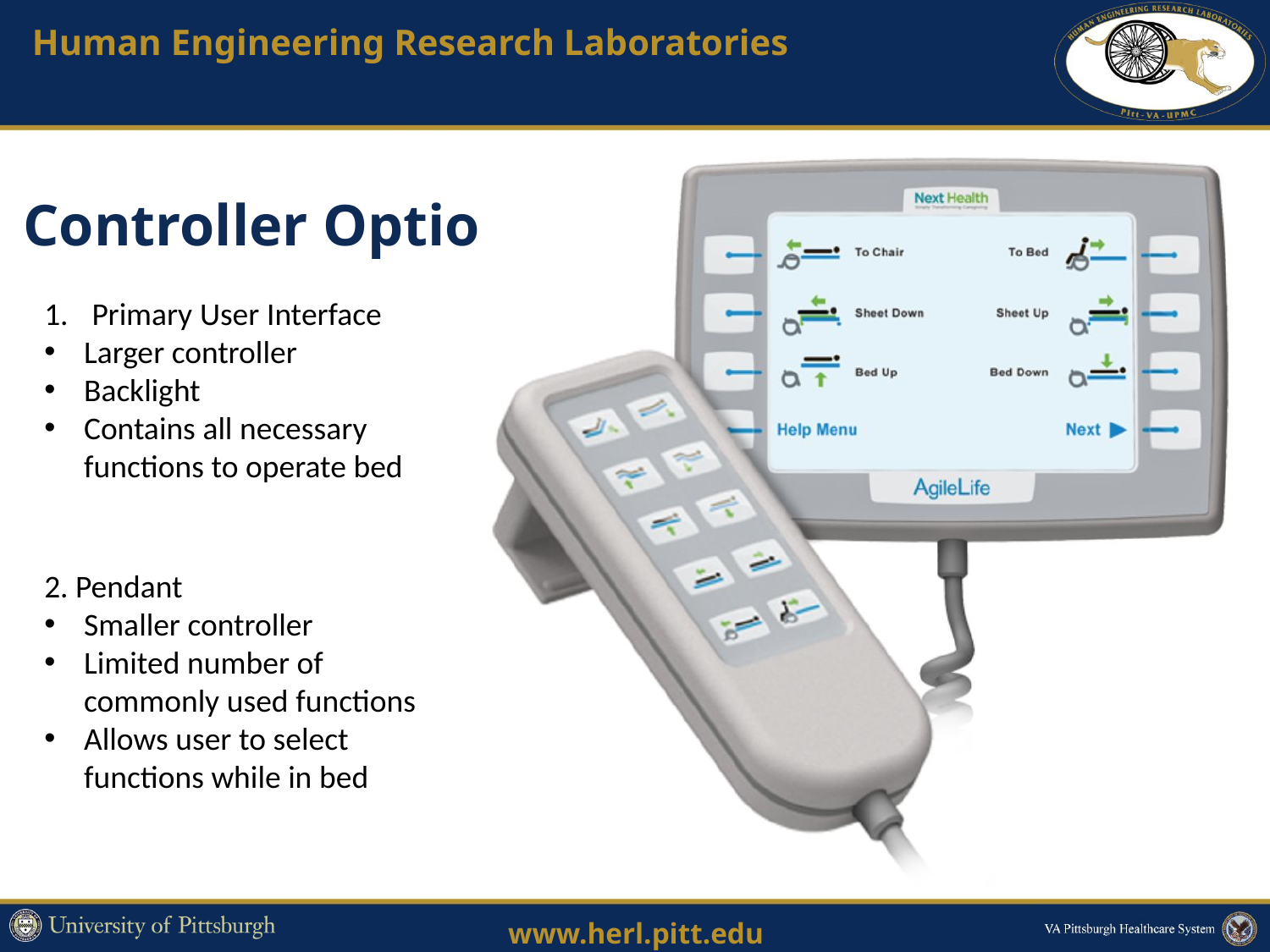

Human Engineering Research Laboratories
Controller Options:
Primary User Interface
Larger controller
Backlight
Contains all necessary functions to operate bed
2. Pendant
Smaller controller
Limited number of commonly used functions
Allows user to select functions while in bed
 www.herl.pitt.edu

## Slide 12
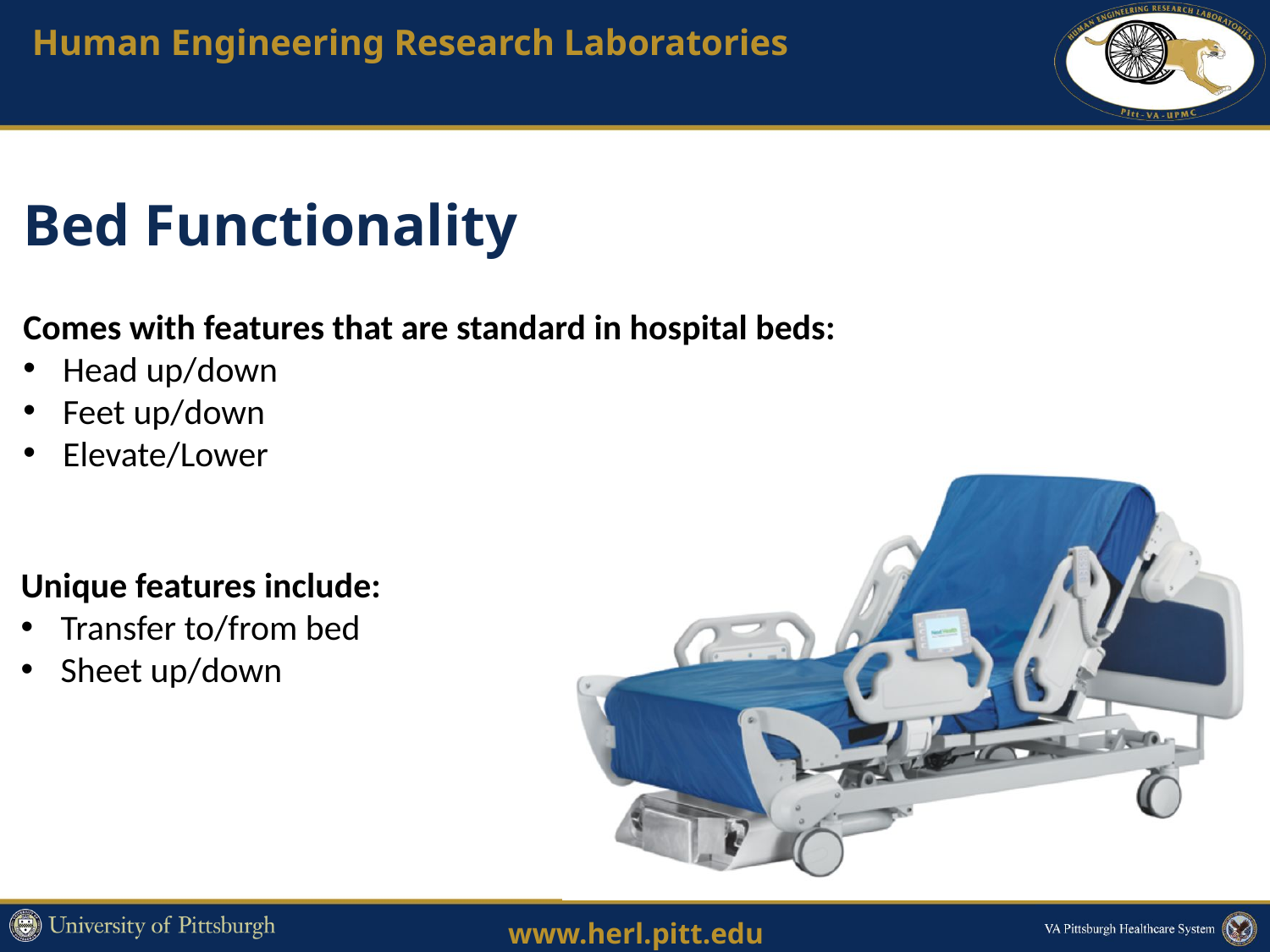

Human Engineering Research Laboratories
Bed Functionality
Comes with features that are standard in hospital beds:
Head up/down
Feet up/down
Elevate/Lower
Unique features include:
Transfer to/from bed
Sheet up/down
 www.herl.pitt.edu

## Slide 13
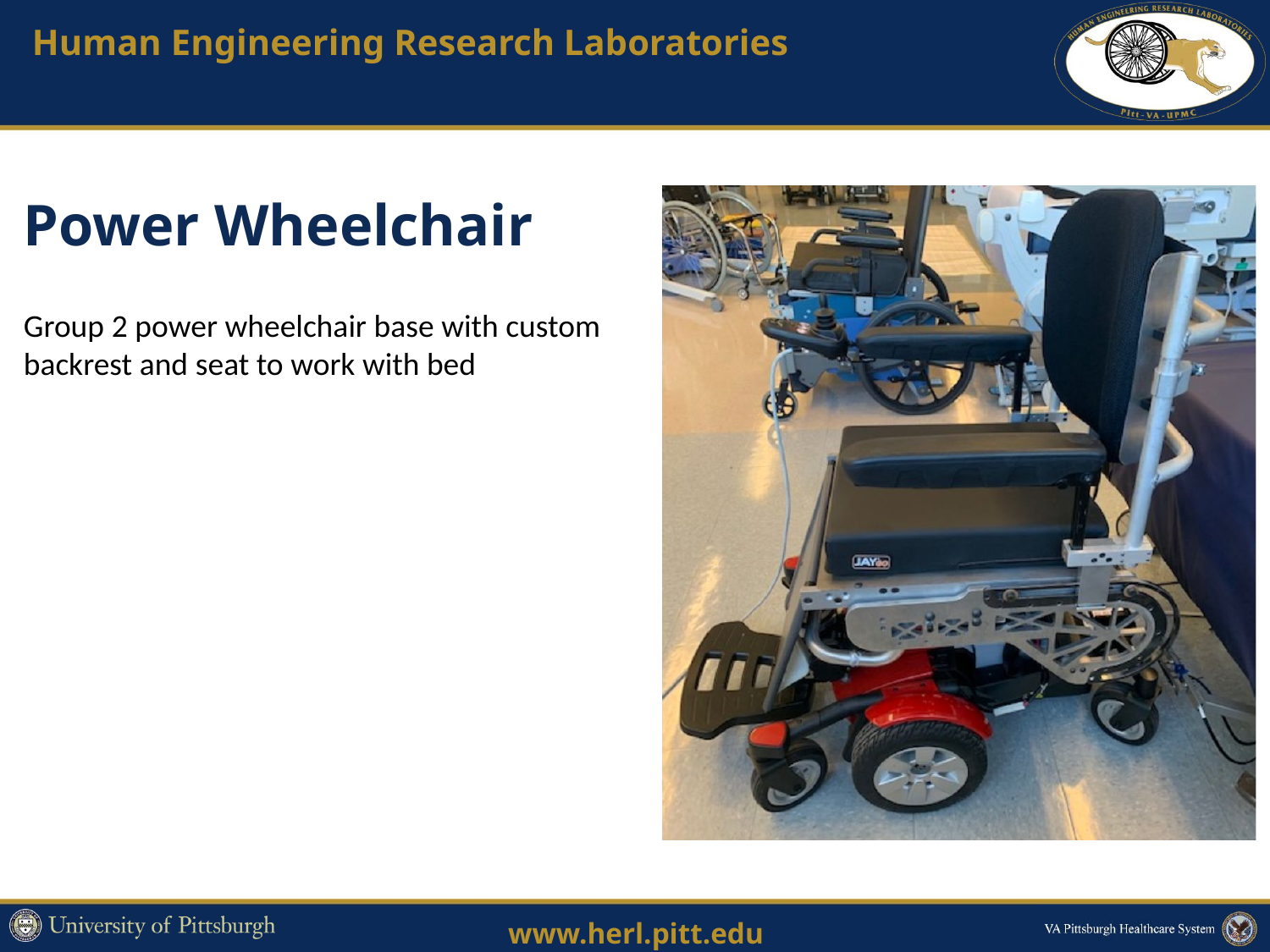

Human Engineering Research Laboratories
Power Wheelchair
Group 2 power wheelchair base with custom backrest and seat to work with bed
 www.herl.pitt.edu

## Slide 14
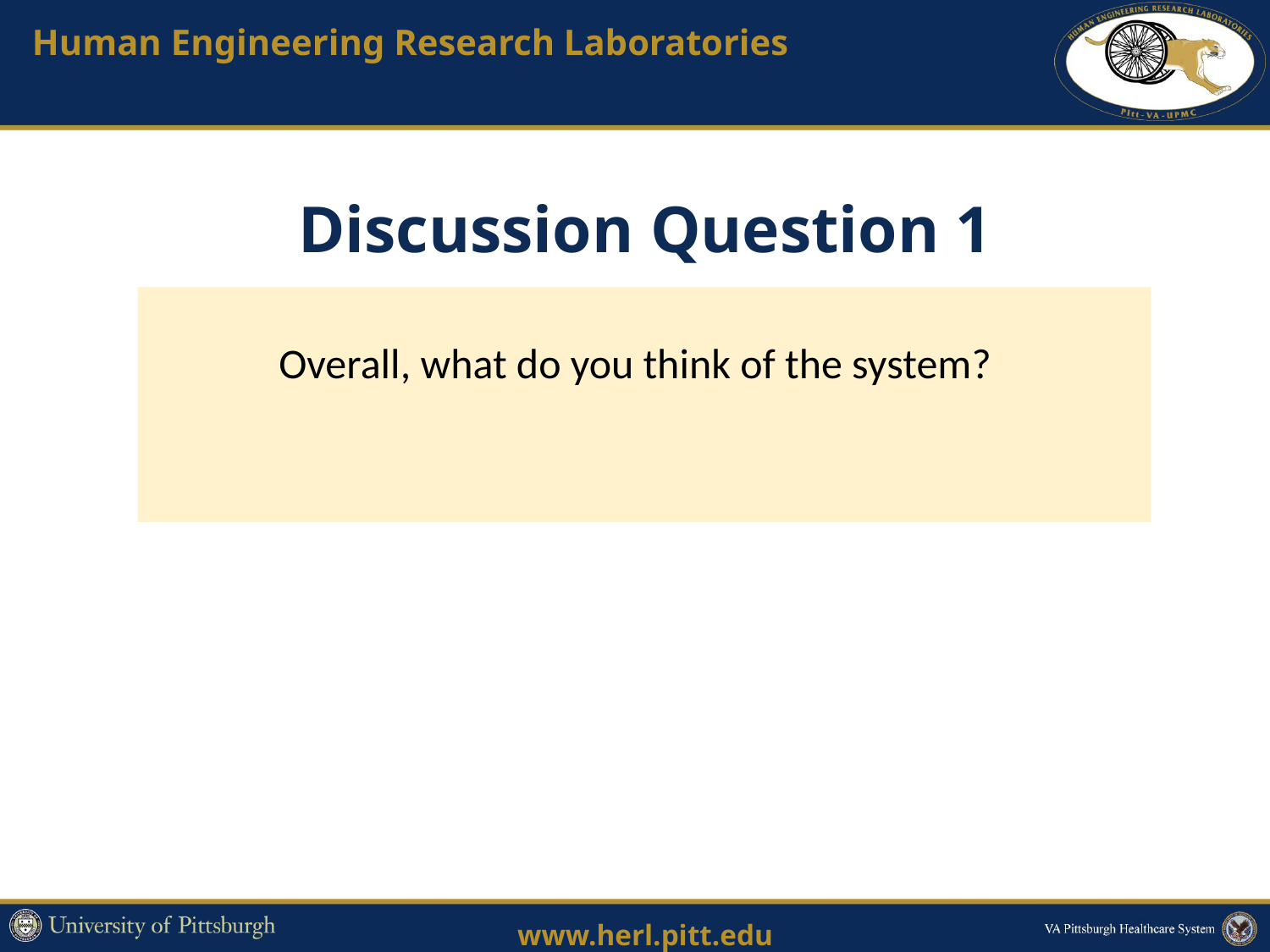

Human Engineering Research Laboratories
Discussion Question 1
Overall, what do you think of the system?
 www.herl.pitt.edu

## Slide 15
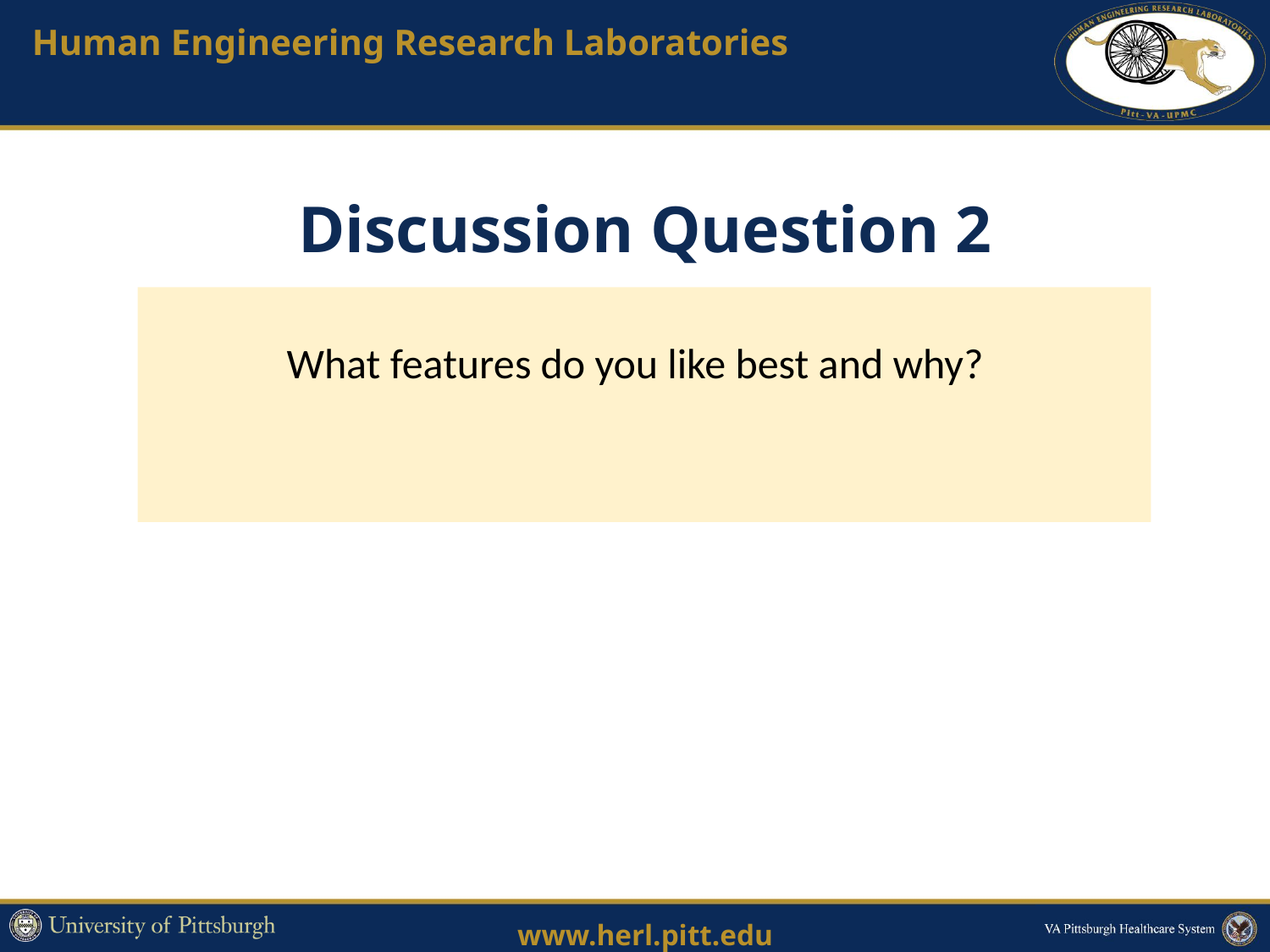

Human Engineering Research Laboratories
Discussion Question 2
What features do you like best and why?
 www.herl.pitt.edu

## Slide 16
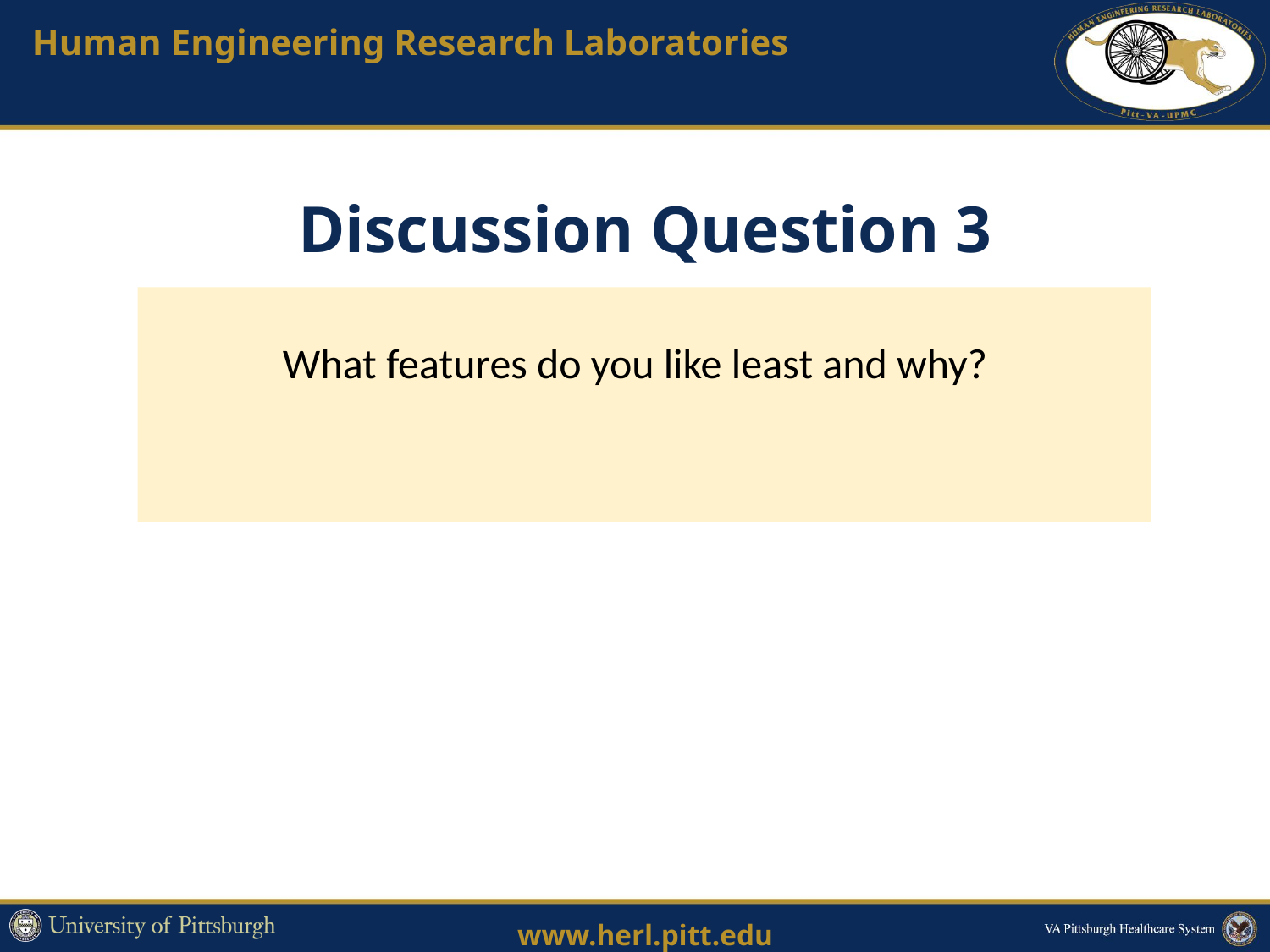

Human Engineering Research Laboratories
Discussion Question 3
What features do you like least and why?
 www.herl.pitt.edu

## Slide 17
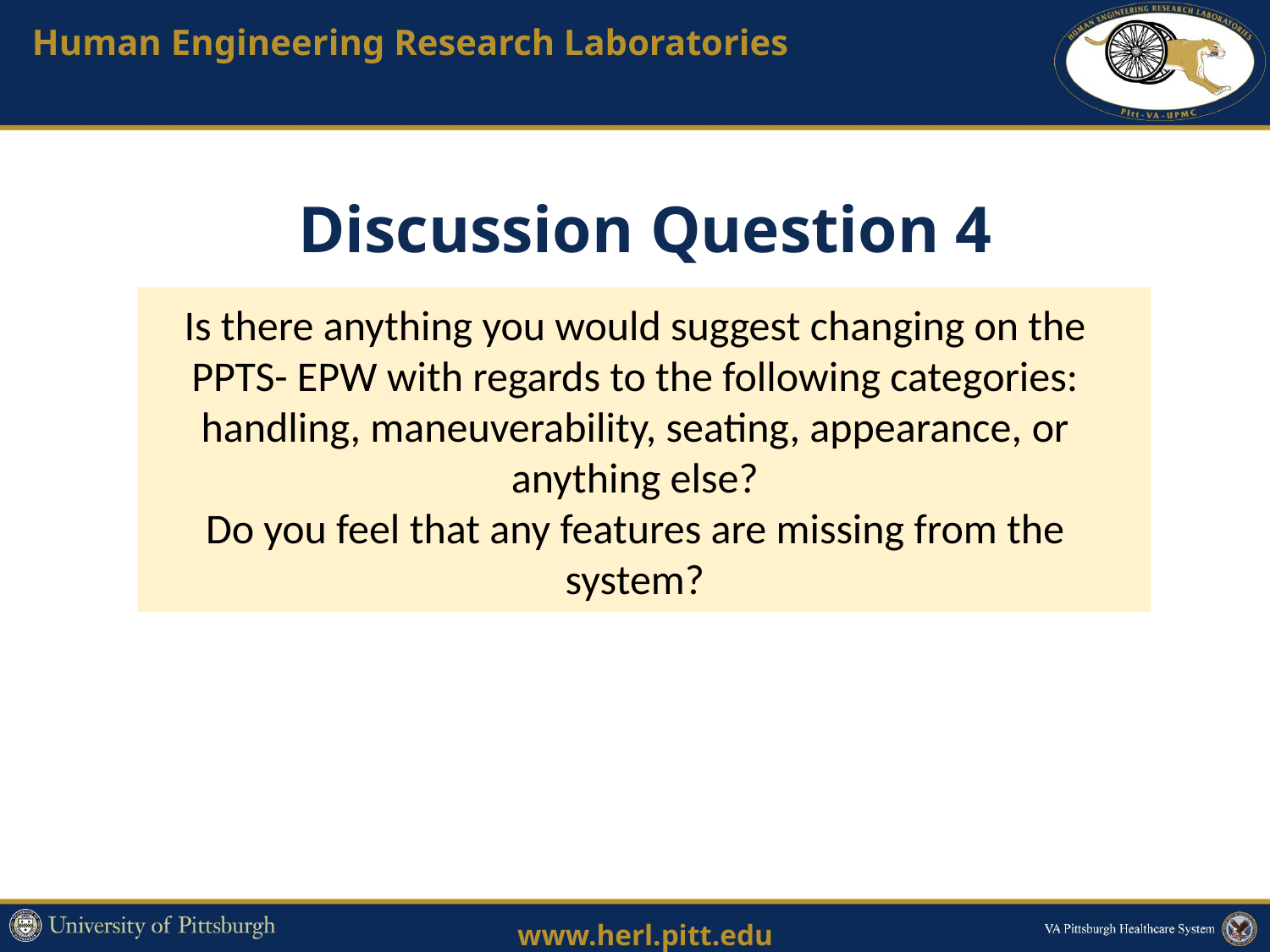

Human Engineering Research Laboratories
Discussion Question 4
Is there anything you would suggest changing on the PPTS- EPW with regards to the following categories: handling, maneuverability, seating, appearance, or anything else?
Do you feel that any features are missing from the system?
 www.herl.pitt.edu

## Slide 18
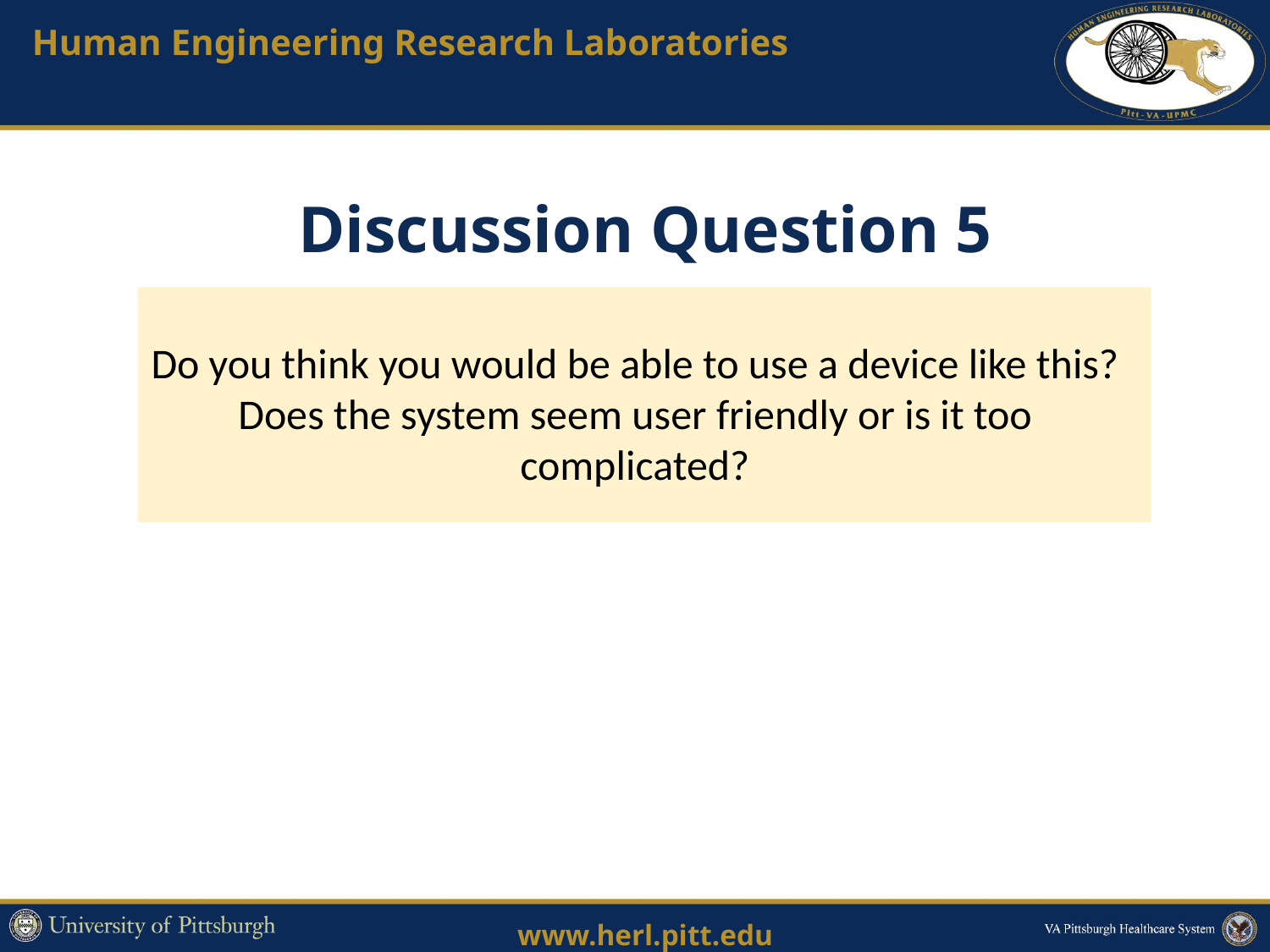

Human Engineering Research Laboratories
Discussion Question 5
Do you think you would be able to use a device like this? Does the system seem user friendly or is it too complicated?
 www.herl.pitt.edu

## Slide 19
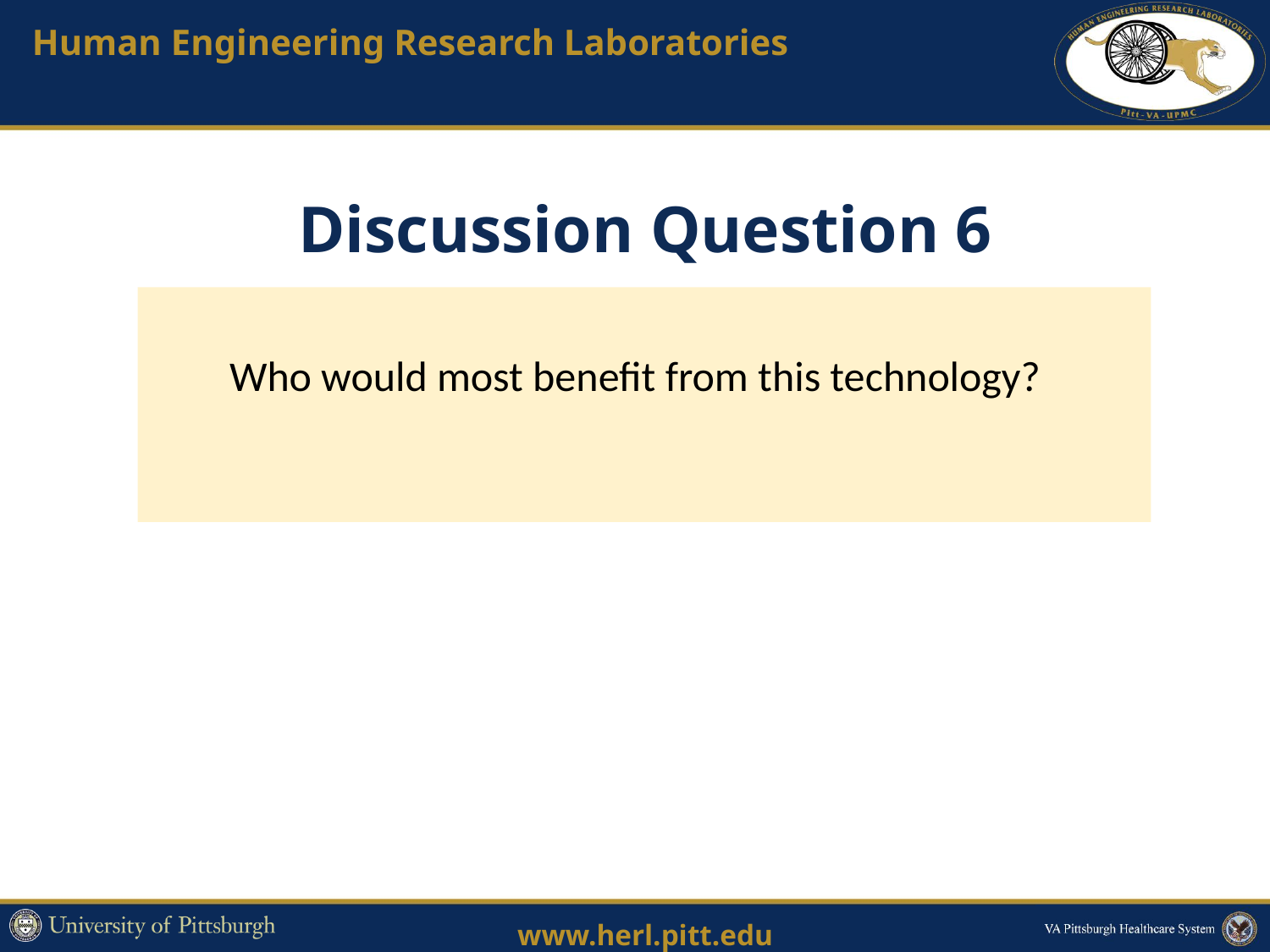

Human Engineering Research Laboratories
Discussion Question 6
Who would most benefit from this technology?
 www.herl.pitt.edu

## Slide 20
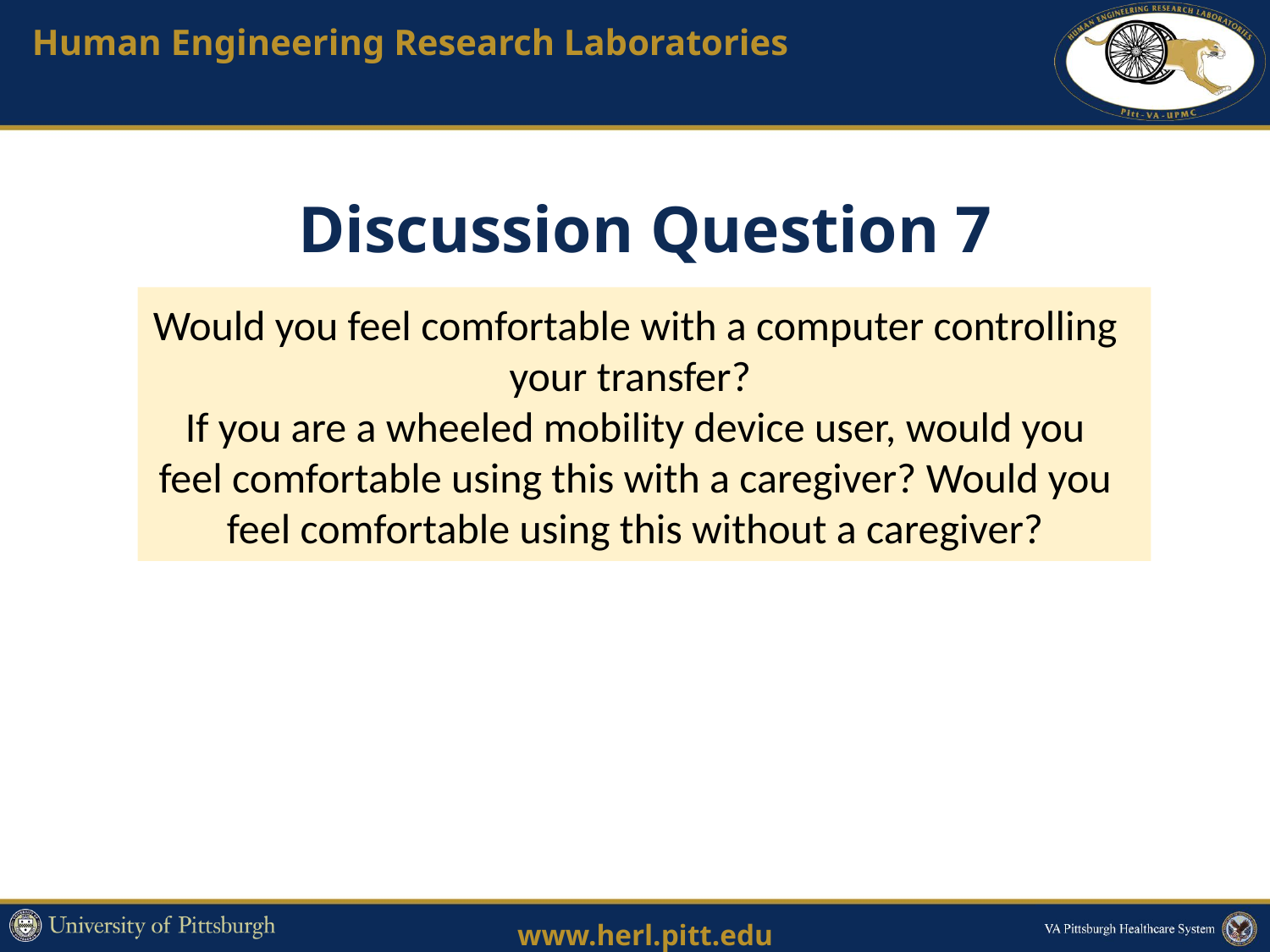

Human Engineering Research Laboratories
Discussion Question 7
Would you feel comfortable with a computer controlling your transfer?
If you are a wheeled mobility device user, would you feel comfortable using this with a caregiver? Would you feel comfortable using this without a caregiver?
 www.herl.pitt.edu

## Slide 21
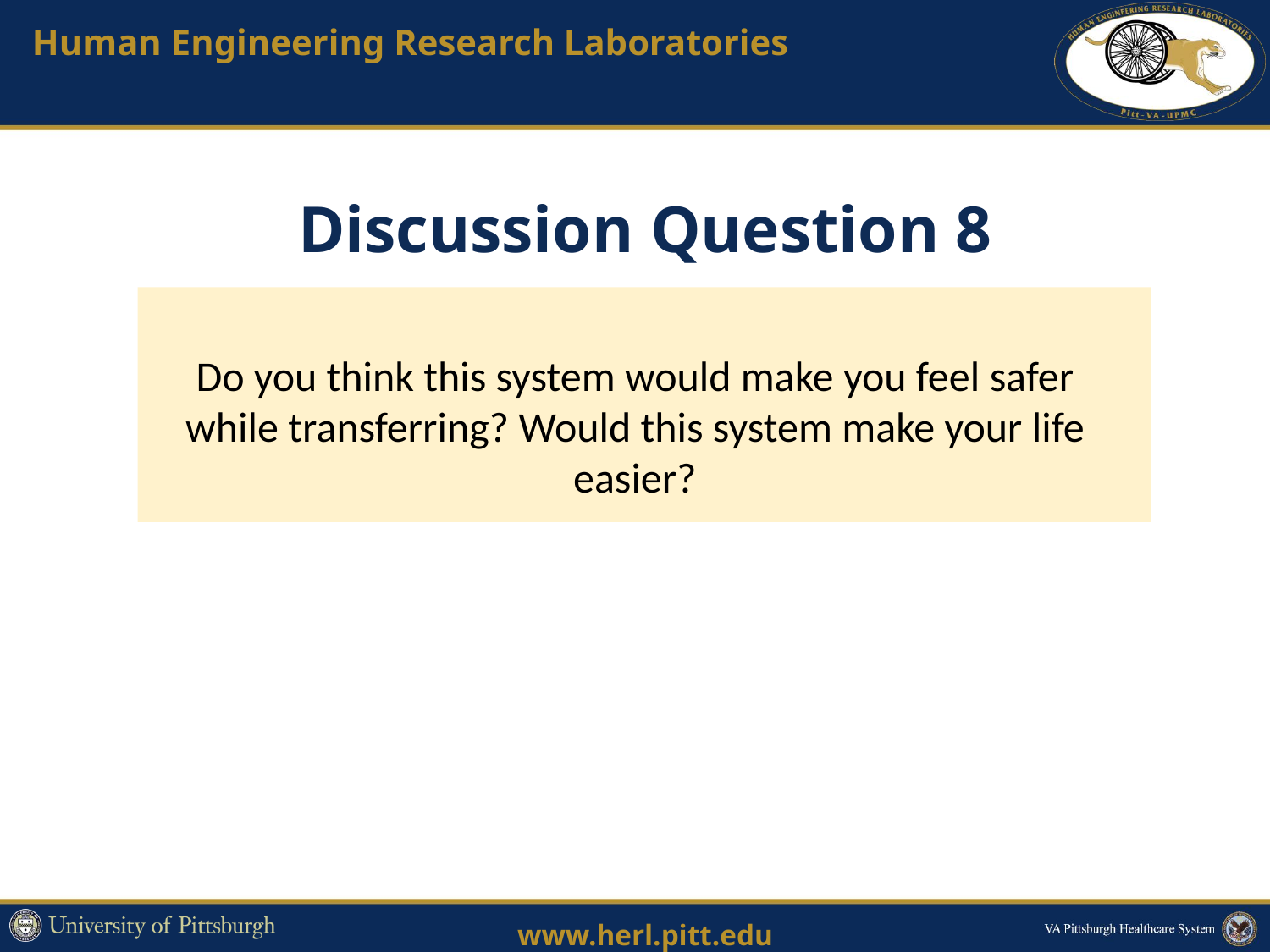

Human Engineering Research Laboratories
Discussion Question 8
Do you think this system would make you feel safer while transferring? Would this system make your life easier?
 www.herl.pitt.edu

## Slide 22
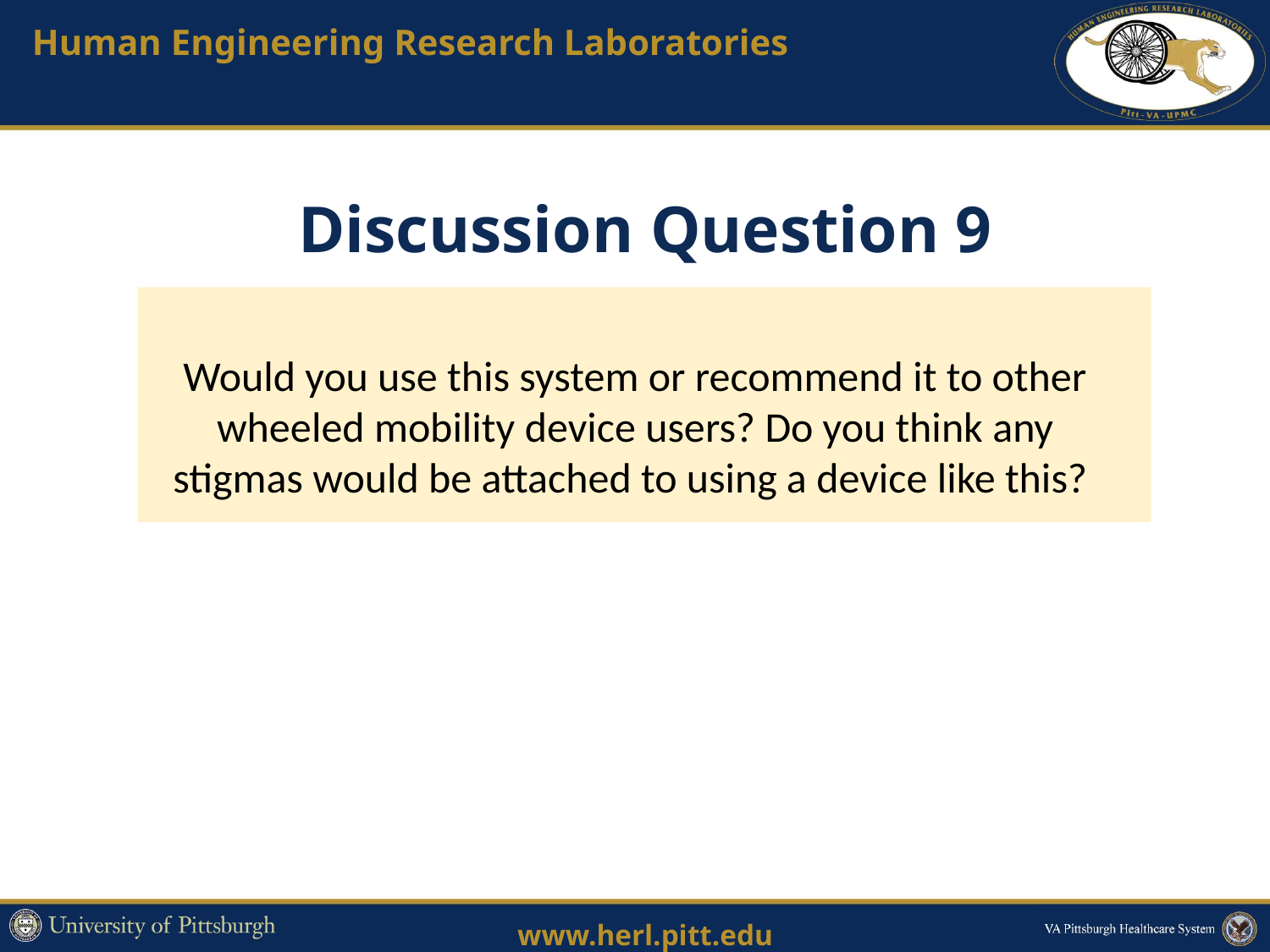

Human Engineering Research Laboratories
Discussion Question 9
Would you use this system or recommend it to other wheeled mobility device users? Do you think any stigmas would be attached to using a device like this?
 www.herl.pitt.edu

## Slide 23
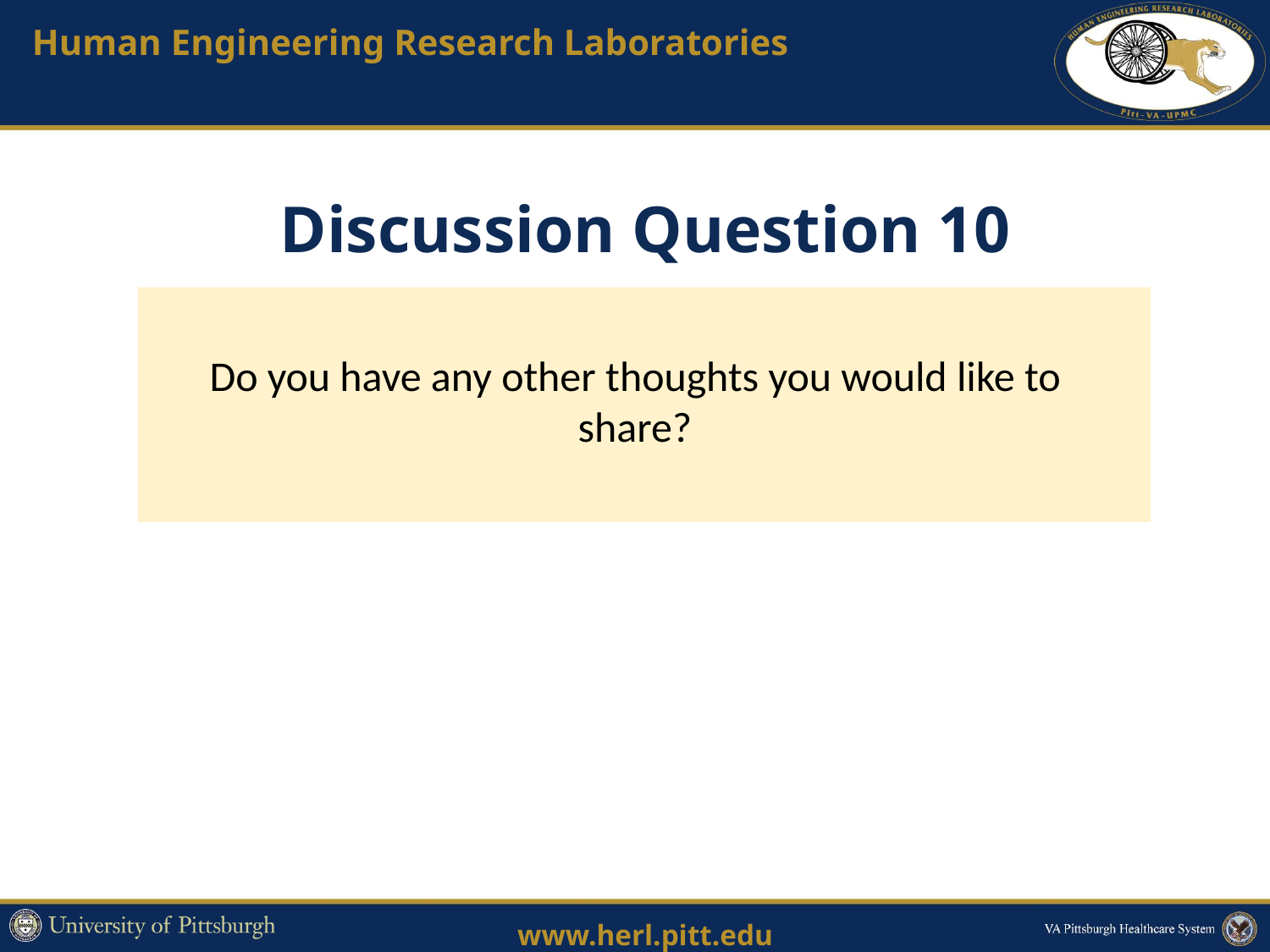

Human Engineering Research Laboratories
Discussion Question 10
Do you have any other thoughts you would like to share?
 www.herl.pitt.edu

## Slide 24
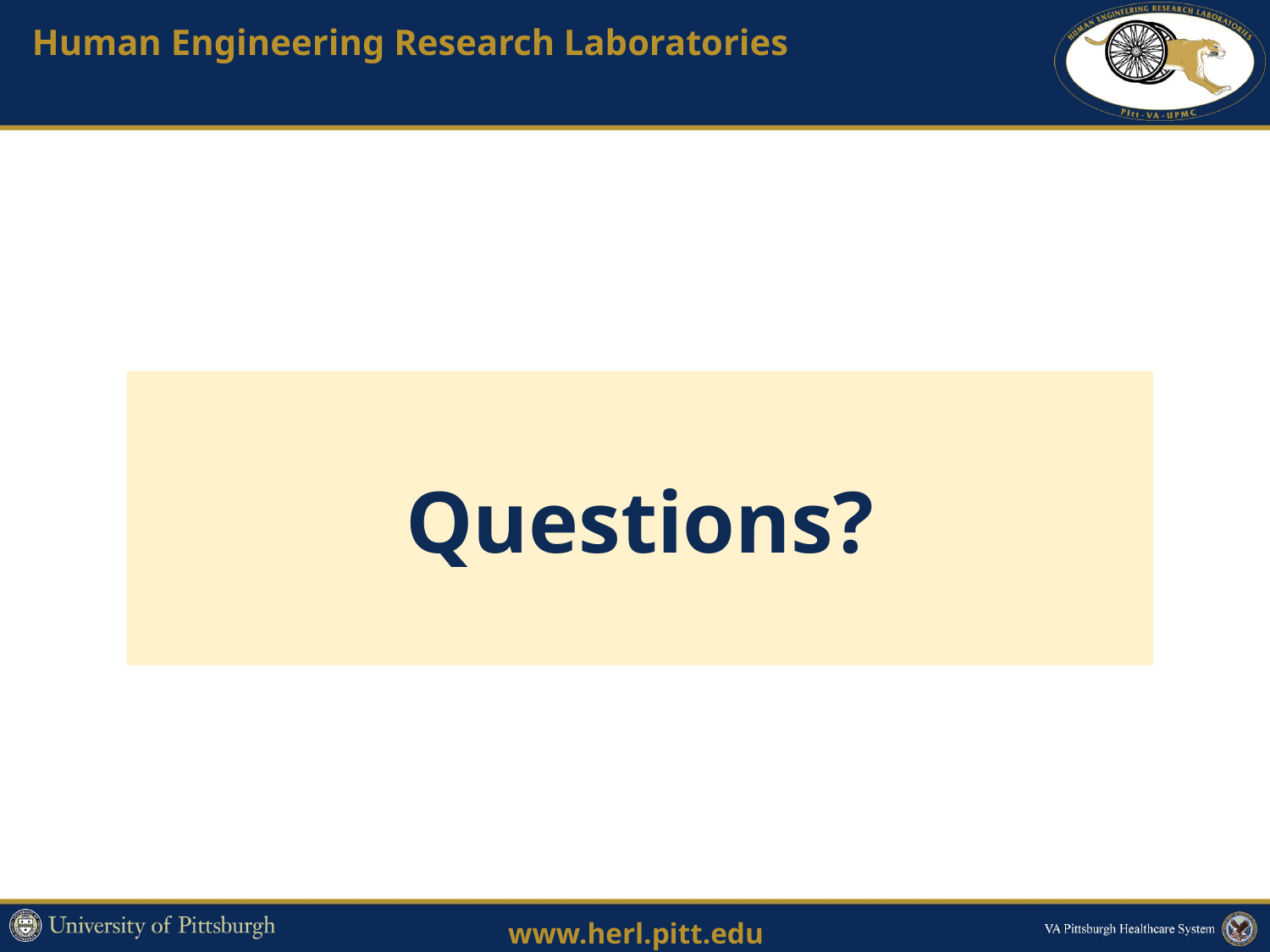

Human Engineering Research Laboratories
Questions?
 www.herl.pitt.edu

## Slide 25
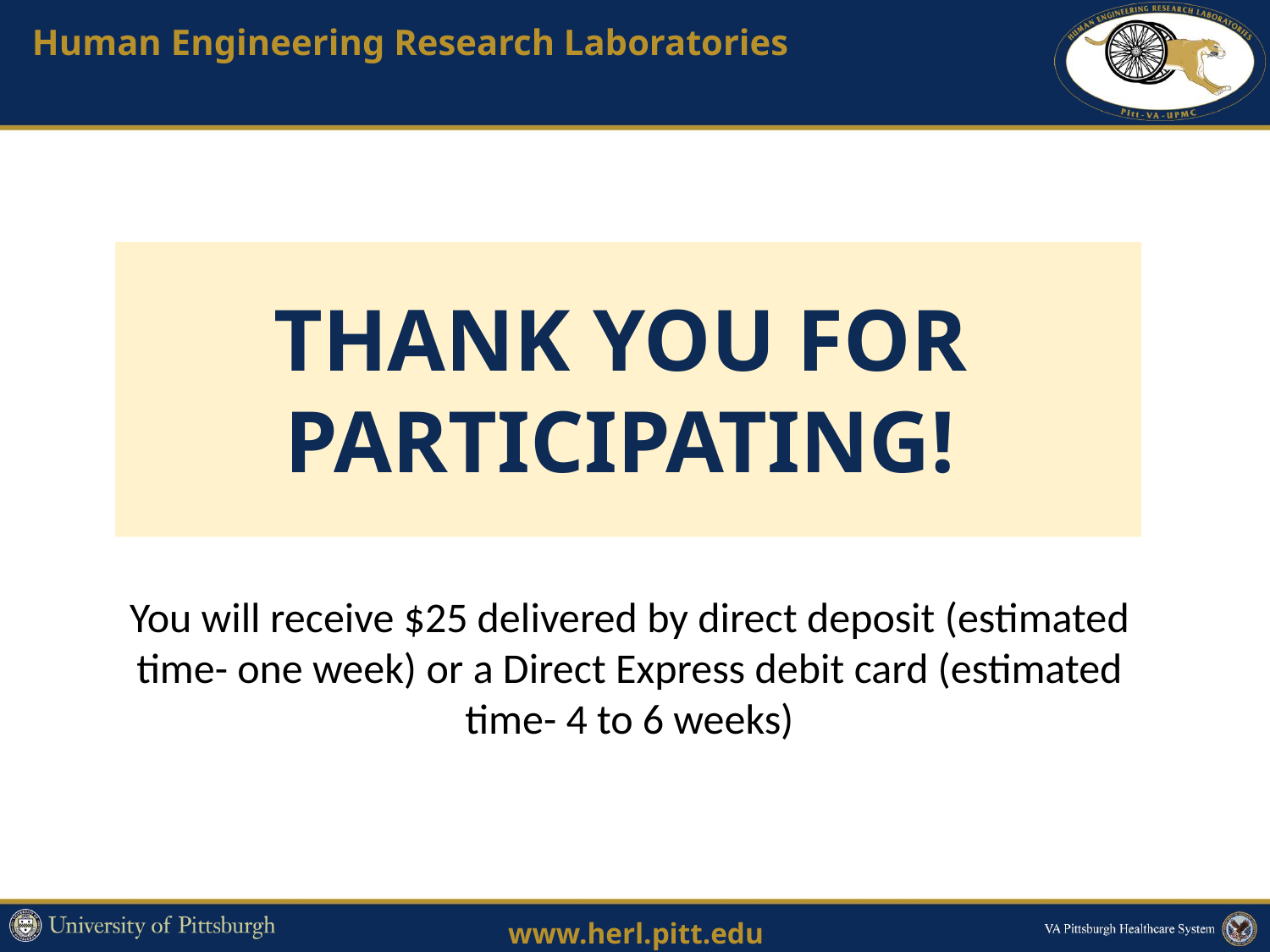

Human Engineering Research Laboratories
THANK YOU FOR PARTICIPATING!
You will receive $25 delivered by direct deposit (estimated time- one week) or a Direct Express debit card (estimated time- 4 to 6 weeks)
 www.herl.pitt.edu

## Slide 26
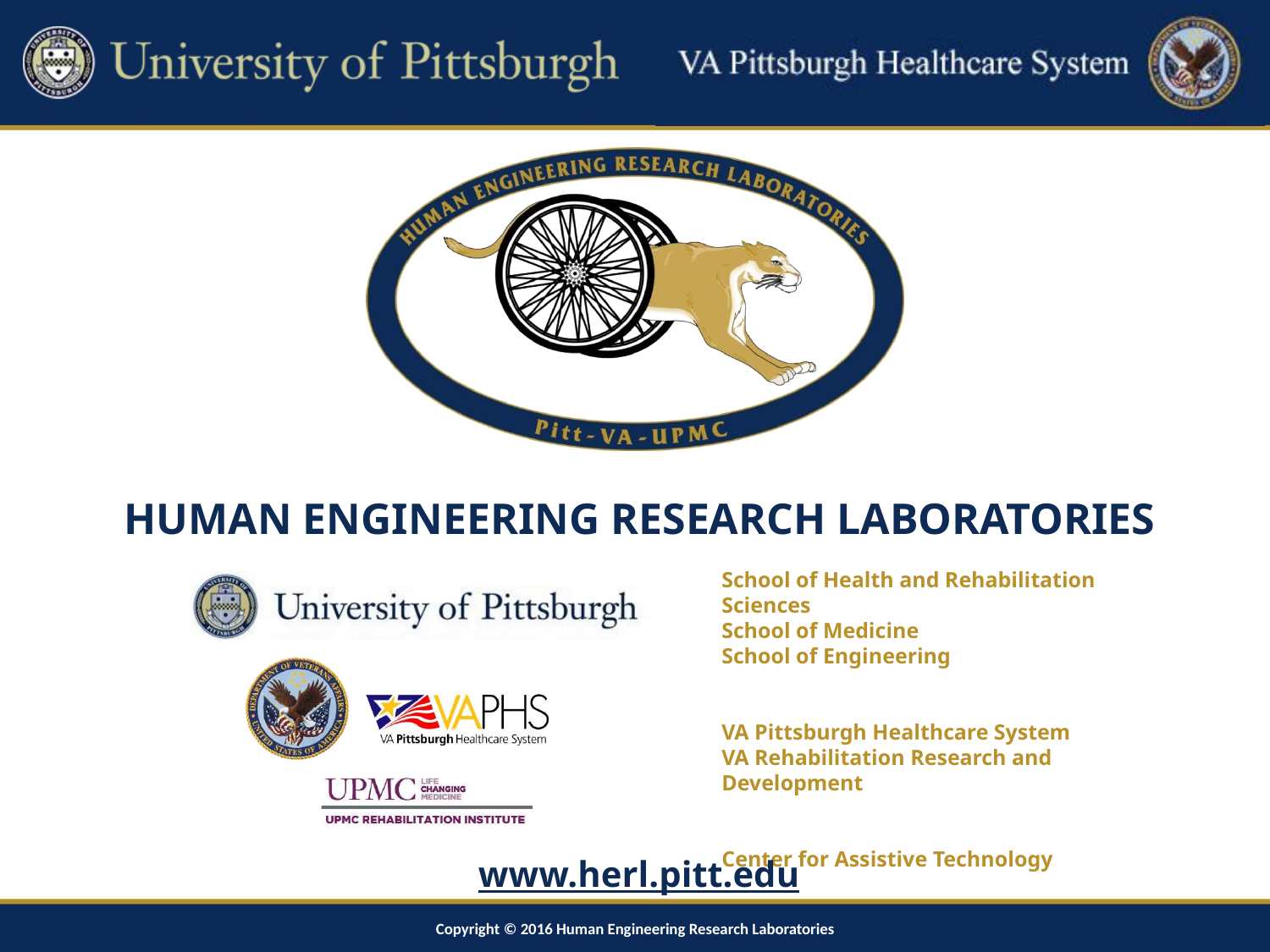

HUMAN ENGINEERING RESEARCH LABORATORIES
School of Health and Rehabilitation Sciences
School of Medicine
School of Engineering
VA Pittsburgh Healthcare System
VA Rehabilitation Research and Development
Center for Assistive Technology
 www.herl.pitt.edu
Copyright © 2016 Human Engineering Research Laboratories
